# Comparative Thermal Performance of Kelp and Herbivores Across a Latitudinal Gradient in Ocean Temperature

**DOI:** 10.1002/ece3.73910

**Published:** 2026-07-06

**Authors:** Claire Butler, Catriona L. Hurd, Adriana Vergés, Scott Ling, Scott Bennett

**Affiliations:** ^1^ Institute for Marine and Antarctic Studies (IMAS), University of Tasmania Hobart Tasmania Australia; ^2^ Centre for Marine Science and Innovation, School of Biological, Earth and Environmental Sciences, University of New South Wales Sydney New South Wales Australia

## Abstract

The interaction between herbivorous sea urchins and their macroalgal food source plays a fundamental role in regulating community structure on temperate reefs across the globe. Water temperature strongly influences the strength of this interaction, through its effects on the rate of metabolic processes. In southeastern Australia, warming temperature has also facilitated the range extension of some urchin species, which are now threatening macroalgal habitats across ∼2000 km of coastline. Despite the importance of temperature in this system, the impact of warming on the relative performance of macroalgae and urchins across their range is unknown. We measured the temperature dependence of respiration rates in two barren‐forming sea urchin herbivores (*Centrostephanus rodgersii* and *Heliocidaris erythrogramma*), and of photosynthetic rates of their macroalgal food source (*Ecklonia radiata*). This was done at 6–9 temperatures ranging from 5°C–35°C at each of six sites in Australia. These sites span these species' latitudinal distributions, covering a latitudinal range of 12° and a temperature range of 8°C. We found clear differences across latitude in the performance of all three species. At warmer latitudes, *E. radiata* showed decreased maximum photosynthetic rates while respiration rates for *C. rodgersii* demonstrated increased sensitivity to acute temperature change. For *H. erythrogramma*, cool‐edge populations showed a plateau in respiration rates at the highest temperature tested, while rates in central and warm‐edge populations continued to increase. Overall these patterns indicate that temperatures at the warm range edge may be approaching upper thermal tolerance limits for all three species, although *C. rodgersii* may have a slightly higher thermal threshold compared to *E. radiata* , and may persist longer in a warming environment. This study provides novel insights into the comparative thermal performance of kelp and urchins across their entire latitudinal distribution and highlights the need to consider different populations of species to understand the impacts of warming on marine ecosystems.

## 1 Introduction

Climate change is causing radical changes to marine ecosystems globally. As temperatures rise, species' abundances, distribution, phenology, and physiological function are changing (Poloczanska et al. 2013). The ecological impacts of these changes are already evident with shifts in the composition and function of ecosystems (Pecl et al. 2017). Understanding what changes will occur is critical for the management of biodiversity and ecosystem services, but predicting how species will respond is challenging. Species' responses to warming depend on myriad factors, including both abiotic and biotic interactions that may change through space and time. Knowledge of species' responses to warming across large spatial and temporal scales is therefore essential to help unravel these complex dynamics and accurately predict climate change impacts.

Latitudinal gradients provide a valuable and interesting means through which to understand temperature‐driven impacts on species and ecosystems. One of the key variables that changes across latitude is temperature, and investigation of how the thermal response of species changes between populations along a latitudinal gradient can provide information on the potential impacts of continued warming (Bennett et al. 2015, 2022; Vergés et al. 2018). For example, a study investigating thermal tolerance of an intertidal crab across a 3000 km latitudinal gradient in Chile found differences in thermal performance that indicated higher latitude populations may be more vulnerable to warming than low latitude populations (Gaitán‐Espitia et al. 2014). Similarly, Wernberg et al. (2010) measured latitudinal differences in metabolic adjustment and recovery from disturbance in a habitat‐forming kelp species in Australia. This revealed decreasing resilience of kelp forests to perturbation in warmer waters, indicating increasing temperatures may lead to persistent loss of this important habitat.

One metric that is well‐suited to assessing species' response to temperature across large spatial scales is metabolic rate. Metabolism is a fundamental biological rate that is comparable between taxa, influences numerous species traits and population processes (Brandl et al. 2023), and provides a logistically feasible metric to measure across large scales. Metabolic rate refers to the sum of biochemical reactions that provide an organism with energy. In animals, metabolic rate is typically estimated by measuring an organism's oxygen consumption via respirometry experiments (Schuster and Bates 2023), while in plants and macroalgae, photosynthetic rate (i.e., oxygen production) is the important metabolic process (Hurd et al. 2014). Like most biochemical reactions, rates of photosynthesis and respiration are directly influenced by temperature, particularly in ectothermic species whose body temperature and thus physiological processes reflect environmental conditions (Hurd et al. 2014; Angilletta et al. 2002). The effect of temperature on photosynthesis can be described by a thermal performance curve, which is characterised by a rising phase as temperature increases, a peak that encompasses the thermal optimum, and a falling phase at higher temperatures. While theory predicts the effect of temperature on respiration conforms to this same shape (Schulte 2015), in practice respiration rates have an analogous rising phase, but will typically increase until a critical thermal maximum is reached and it is not often possible to measure a falling phase (e.g., Healy and Schulte 2012; Norin et al. 2013; Vernberg 1959; Schulte et al. 2011; Lemoine and Burkepile 2012; Scholander et al. 1953). This has important implications for interpretation. If the peak of a respiration thermal performance curve corresponds to a critical or lethal limit, rather than a thermal optimum, caution is required to avoid overestimating thermal limits.

Although the thermal performance curve describes the general relationship between temperature and the rate of photosynthesis/respiration, there is substantial variation in its shape and position. For example, different taxa may have different metabolic demands depending on their phylogenetic history and contemporary distribution (Clarke and Johnston 1999). Thermal performance curves may also differ among life‐history stages (Dahlke et al. 2020). Importantly, phenotypic plasticity and/or adaptation to local environmental temperatures can also cause variation in its shape and position, resulting in different patterns of thermal performance across latitude in ectothermic species (Schulte et al. 2011; Stillman 2004). For species acclimated to their local environment, or where reduced population connectivity allows for local thermal adaptation, thermal performance curves of different populations may shift horizontally along the x‐axis such that optimum temperatures reflect local temperature regimes (Bennett et al. 2015; Gardiner et al. 2010). In some cases vertical shifts in the performance curve have also been recorded (i.e., differences in the maximum rate at the thermal optimum; (DeLong et al. 2018)). In contrast, for species that do not acclimate, or where high gene flow prevents adaptation, no differences in the shape or position of a thermal performance curve are expected (Gardiner et al. 2010; Dunphy et al. 2012).

When considering the ecological impacts of warming, another important aspect to examine is the comparative performance of interacting species. How species perform relative to their competitor, predator, or prey is of critical importance, as changes to these interactions may not only impact the species in question, but in some cases can alter whole‐ecosystem function and resilience (Kordas et al. 2011). For example, in marine ecosystems, herbivorous urchins and fishes can play a key role in mediating the abundance of macroalgae (Poore et al. 2012). If warming favours the physiological performance of herbivores relative to their macroalgal food source, the impact of herbivores may increase, potentially leading to overgrazing. This can have catastrophic consequences and in some cases may cause regime shifts (Vergés et al. 2014; Ling et al. 2015).

Across the eastern Great Southern Reef (*sensu* (Bennett et al. 2016)) in Australia, two dominant urchin herbivores, *Centrostephanus rodgersii* and *Heliocidaris erythrogramma*, play a fundamental role in mediating the abundance of the golden kelp (*Ecklonia radiata*, order Laminariales; Ling and Keane 2024). Each species has different thermal affinities and life history traits which may influence their population connectivity and response to warming across their latitudinal distribution. *Centrostephanus rodgersii* is a warm affinity species that is native to the warm‐temperate waters off the southeast coast of mainland Australia. However, in recent decades, climate‐driven warming and strengthening of the East Australian Current (Ridgway and Ling 2023), coupled with this species' long larval phase (40 days–4 months; Huggett et al. 2005; Mos et al. 2020), have facilitated its range extension to Tasmania's east and southeast coasts (Ling 2008). In contrast, *H. erythrogramma* is distributed across the entire Great Southern Reef but has a cooler affinity and higher abundances in more southern waters. It has a short larval phase (3–5 days; Williams and Anderson 1975), which contributes to low genetic connectivity between different populations (McMillan et al. 1992). Similarly, and like many macrophytes (King et al. 2018), populations of *E. radiata* are known to be genetically isolated across its range (Coleman et al. 2009, 2020), which extends across the entire Great Southern Reef. Within this region, populations of *E. radiata* are typically more abundant in cool‐temperate waters (Wernberg et al. 2010).

To understand how warming will impact the comparative performance of kelp and urchins across their range in Australia, we examine thermal performance curves of photosynthetic rates of the golden kelp, *E. radiata* , and respiration rates of two urchin species (*C. rodgersii* and *H. erythrogramma*), between populations across a latitudinal gradient in temperature that spans each species' thermal distribution. We hypothesised that (1) thermal performance of *E. radiata* and *H. erythrogramma* will differ between populations, indicating acclimation or adaptation to local temperatures and reflective of their relatively low dispersal capacity and population connectivity, (2) thermal performance of *C. rodgersii* will be similar across latitude, consistent with their long larval phase and high population connectivity, and (3) based on thermal affinities, *E. radiata* and *H. erythrogramma* will have lower thermal limits relative to *C. rodgersii*.

## 2 Methods

### 2.1 Study Sites

**FIGURE 1:**
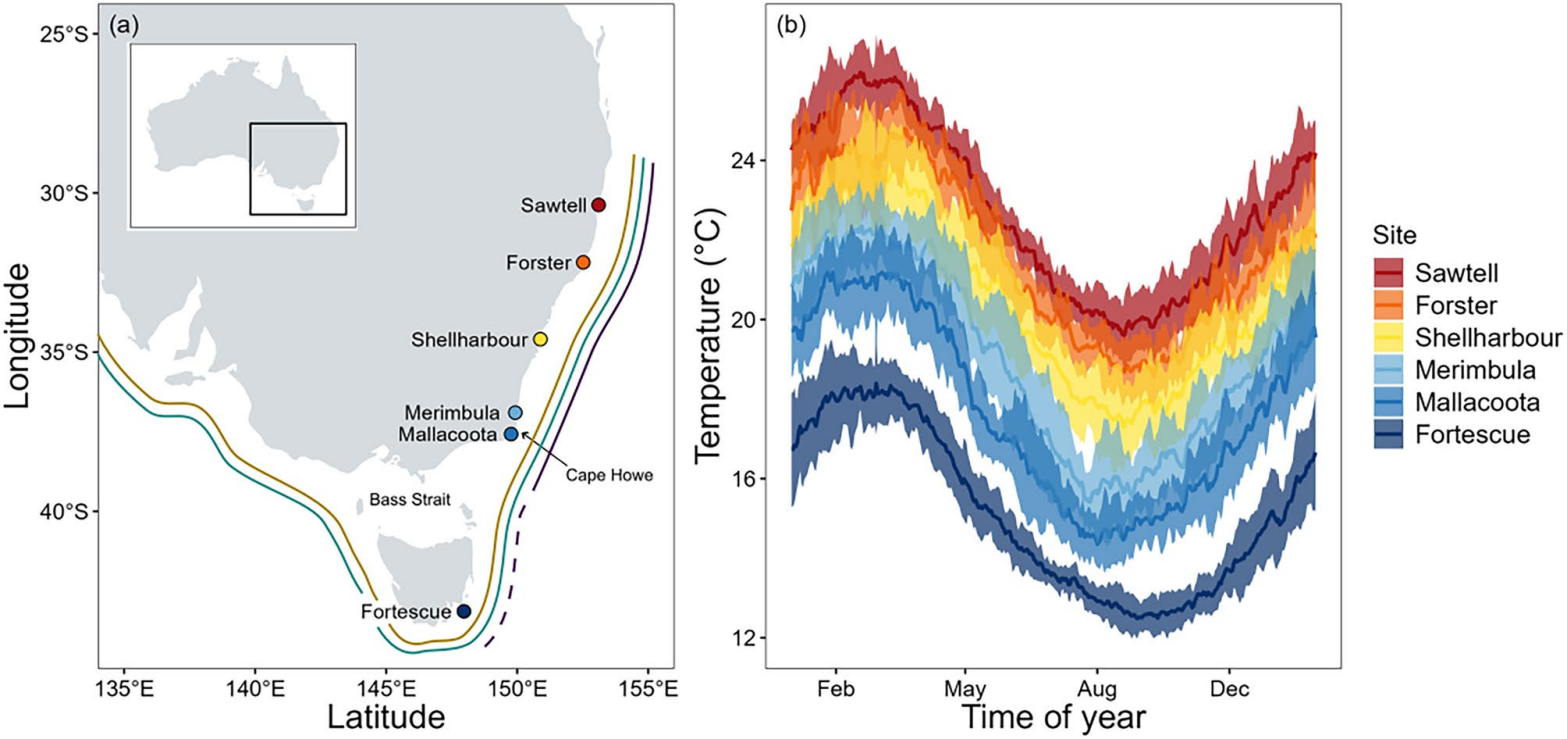
(a) Map of experimental sites including the distribution of *E. radiata* (brown solid line), *H. erythrogramma* (green) and *C. rodgersii* (purple). For *C. rodgersii* the dashed line represents its extended range. (b) annul temperature cimatology at each site based on mean daily temperatures averaged for the period 2012–2023. Shaded areas represent the standard deviation in daily temperatures between years.

Experiments were carried out at six sites spanning the latitudinal distribution of each of the three species ( *E. radiata* , *H. erythrogramma*, *C. rodgersii*) on the southeast coast of Australia (Figure 1). Ocean conditions in this area are dominated by the flow of the East Australian Current, a warm, western boundary current that flows from north to south along Australia's southeast coast and generates a strong latitudinal gradient in ocean temperature (Kerry and Roughan 2020). The six sites—Sawtell (−30.38°, 153.11°), Forster (−32.18°, 152.52°), Shellharbour (−34.59°, 150.89°), Merimbula (−36.90°, 149.93°), Mallacoota (−37.57°, 149.76°), and Fortescue Bay (−43.14°, 147.97°)—range from the northern‐ to southern‐most distribution limits for each species, with intermediate sites spaced at approximately 1°C–2°C intervals of ocean temperature where possible. Local site selection also took into account the comparability of habitat types. Sites were chosen on exposed to semi‐exposed sections of open rocky coastline with medium to dense cover of canopy‐forming species and where *E. radiata* constituted a dominant species. Sites were visited sequentially from Sawtell to Fortescue Bay over a seven‐week period in the summer and early autumn (Feb–March) of 2023. By visiting sites sequentially, we aimed to sample at the time of peak ocean temperature in each location. Although gonad size is not expected to influence urchin respiration rates (Giese et al. 1966), the seasonal timing of this work none‐the‐less avoided the spawning season for both urchin species (Jun–Aug for *C. rodgersii* (Ling et al. 2008) and Dec–Jan for *H. erythrogramma* (Keesing 2020)), and no urchins were observed to spawn during experiments.

### 2.2 SST Climatology

Mean daily satellite‐derived sea surface temperature (SST) data for each site were extracted from the NOAA National Climate Data Centre Optimally Interpolated AVHRR product at a 1/4° grid resolution (Optimum Interpolation Sea Surface Temperature, www.ncdc.noaa.gov/oisst). A mean annual climatology was calculated for each site using daily temperatures averaged across the 12 year period between 2012 and 2023. The mean temperature for the 30 days prior to specimen collection at each site was also calculated.

### 2.3 Specimen Collection

Sea urchin and macroalgal samples were collected by hand on SCUBA at each location, from depths of < 5 m (for *H. erythrogramma*) and 2–10 m (for *E. radiata* , and *C. rodgersii*). For urchins, those with a test diameter over 80 mm were targeted at each site, however in locations where this size class was not found, the largest urchins available were collected. For the mean wet weights of urchins collected at each site, see Table S1. For *E. radiata* , second stage juveniles were collected, which showed small and simple side branches (Kirkman 1981). Upon collection, all specimens were transported a short distance back to the temporary field station (undercover outdoor laboratory at accommodation) in sealed barrels of local seawater. Here, they were placed into aerated holding tanks (68 L Nally bins) and maintained at ambient temperature until they were used in metabolic trials within 24–72 h.

### 2.4 Metabolic System

We used a custom‐built experimental system to measure photosynthesis of seaweeds and respiration of urchins (Figure S1). The system consisted of ten acrylic respiratory chambers, each with a volume of 4.5 L, which sat on an aluminium frame housed within a 350 L water bath. Temperature control was achieved using an IKS Aquastar control panel attached to four 600 W Schego titanium heaters and three water chillers (two Teco TK 2000 and one Icemaster G20 glycol chiller). Several aquarium water pumps were placed in the bath to ensure water temperature was uniform throughout. Seawater within the bath was continuously aerated with four air pumps (Aqua One 7500) and ceramic stones. Each chamber was fitted with an internal water pump (Sicce Syncra Nano), PreSens temperature probe (Pt100), and an oxygen sensor spot (SP‐PSt3‐NAU‐D5‐YOP) aligned to a fibre optic cable. Oxygen spots were calibrated using air‐ and nitrogen‐saturated, room temperature seawater.

For measurements of photosynthesis, four LED lights (Zeus‐70 Ledzeal) were installed above the bath. The lights, representing the full underwater spectrum, were installed at the same height and in the same order at each site and set to specific % intensities (80–25–25–80), determined prior to fieldwork to ensure even light intensity across the entire bath. The whole set‐up (bath + lights) was covered in a black tarpaulin to prevent additional daylight altering these standardised conditions.

### 2.5 Experimental Methods

To measure photosynthetic rates of *E. radiata* , nine kelps were selected the evening prior to experiments and manually cleaned of any epiphytic growth and other organisms residing on their lamina and holdfasts. An R‐clip was slipped over the stipe to act as a weight, and the samples were returned to the holding tank overnight. Photosynthetic rates were assessed at multiple temperatures throughout a single day, using a temperature ramping protocol similar to Schuster et al. (2022). Briefly, the water bath containing all ten chambers was chilled overnight to a temperature of 5°C. At the beginning of the day, the nine pre‐selected juveniles were transferred from the holding tanks into a chamber and the LED lights switched on. The tenth specimen‐free chamber acted as a control to account for any background respiration. After one hour of acclimation time, the chambers were closed and a metabolic experiment began. Measurements of temperature and oxygen concentration were made every 4–30 min (depending on the rates of change), using a Presens Fibrox 4 oxygen metre, until a steady increase in oxygen content of at least 5%–10% had been observed. At this point, chambers were reopened and, with juveniles remaining inside each open chamber, the temperature of the water bath was increased to the next target temperature. This was repeated for 7–9 temperature treatments, with a standard 1 h period between treatments in which the next target temperature was reached. Following these methods, each of the nine juveniles was subject to metabolic experiments at increasing temperatures until 30°C, where experiments were stopped. Target temperatures varied between locations in order to best capture the peak of the thermal performance curve (see Table S2 for exact treatment temperatures at each site). Although the temperature ramping rate during these experiments was well above those experienced in natural systems (Spillman et al. 2021), the aim of this work was to compare relative sensitivity of populations across latitude rather than quantify absolute thermal thresholds to specific climate scenarios. Following the completion of the metabolic experiment at the highest temperature, each juvenile was patted dry and weighed.

To measure respiration rates in urchins, nine of the largest individuals of either *C. rodgersii* or *H. erythrogramma* were selected from the holding tanks and placed into a chamber. Mean wet weight (g ± SD) across all sites was 430 ± 151 for *C. rodgersii* and 183 ± 91.1 for *H. erythrogramma*. The same experimental protocol was followed as for *E. radiata* , however, for urchins, measurements of temperature and oxygen concentration were made every 4–30 min until oxygen levels had declined no more than 80% and metabolic experiments were tested at increasing temperatures until the urchin died. Death was considered the point at which urchin spines were drooped, urchins did not respond to stimuli, coelomic contents had been ejected and jaws were slack. After the final metabolic experiment was complete, individuals were removed from the chambers, weighed, and an individual volumetric displacement was measured (volumetric displacement = water volume with urchin—water volume without urchin).

Dates of experimental work for each species and location are listed in Table S3. All specimens were disposed of appropriately after experiments were complete.

### 2.6 Data Analysis

Photosynthetic rates of *E. radiata* and respiration rates of *H. erythrogramma* and *C. rodgersii* were modelled using generalised linear mixed models in the *glmmTMB* package. Models included a second order polynomial term on temperature and a temperature*site interaction. We used a second order polynomial because the unimodal shape meets expectations for the unimodal shape of a thermal performance curve. A random effect of replicate nested within site was also included to account for the repeated measures on replicate individuals. Models were fit with Gaussian distribution unless otherwise stated below.

For photosynthetic rates, the maximum rate (R_*max*_) of photosynthesis and the optimum temperature (T_*opt*_) for photosynthesis were calculated by extracting the maximum rate from the model‐predicted values, and the temperature at which this occurred. Standard errors were derived by parametric bootstrap. Latitudinal trends in photosynthetic performance ($R_{\max}$ and $T_{\mathrm{opt}}$) were assessed by meta‐regression using bootstrapped parameter estimates and their variance. Meta‐regression allowed for estimates to be weighted by their variance (which increased towards warmer waters, particularly for $T_{\mathrm{opt}}$), such that estimates with larger variance were given less weight. Due to the small number of sites across latitude, we conducted a sensitivity analysis to assess the influence of individual sites on the meta‐regression results. This revealed Merimbula to exert a strong influence on both $R_{\max}$ and $T_{\mathrm{opt}}$. To ensure these sites did not unduly influence latitudinal trends, we re‐ran each model after removing this site. Excluding this site did not alter the shape or statistical significance of the latitudinal trend for either $R_{\max}$ or $T_{\mathrm{opt}}$ (Figures S2–S4; Table S4). Because *E. radiata* at Sawtell recorded no net gain in $O_{2}$, this site was removed from meta‐regression analyses and related figures.

For urchin respiration, polynomial models were fit to both mass‐standardised and mass‐independent respiration rates. Mass‐standardised rates were adjusted for the wet mass (g) of the given organism by dividing absolute respiration rate by wet mass. Mass‐standardised models were fit using a Gamma distribution with a log link. Mass‐independent rates were calculated by regressing absolute respiration on individual body mass and extracting the residuals (Bennett 1987). For each species, a generalised linear model of the log of respiration rate was fit against wet weight using natural splines with 2 degrees of freedom and a Gaussian distribution. Only mass‐independent results are presented in the main manuscript, as these appropriately deal with the influence of body mass on respiration rates. Mass‐standardised models show similar patterns, and are presented in the Supporting Information only (Figures S5 and S6; Tables S5–S8). For each model, the significance of fixed effects was assessed using Wald chi‐squared tests. Pairwise comparisons of predicted respiration rates between each site were carried out using *emmeans* at three discrete temperature points—the minimum, median and maximum—across the shared experimental temperature range for each species. For *C. rodgersii* these temperatures were 7.62°C, 18.4°C, 29.6°C. For *H. erythrogramma* comparisons were carried out at 6.42°C, 17.7°C, 29.2°C. All models, including those used to calculate mass‐independent respiration rates, were inspected to ensure they met model assumptions of homogeneity of variances and normality of residuals and transformations used when applicable.

## 3 Results

### 3.1 Temperature Regimes at Experimental Sites

Average daily sea surface temperatures across the study region spanned a range of 8°C over 12° of latitude. Temperatures ranged from 12.5°C–26.2°C (winter minimum at Fortescue and summer maximum at Sawtell respectively, as calculated from the mean annual climatology; Figure 1). Seasonal variation in temperature was similar at all sites, with approximately 6°C–7°C between winter and summer temperatures. The coolest winter temperatures typically occur in late winter and early spring (Aug/Sept) and warmest summer temperatures in late summer and early autumn (Feb/March), when sampling took place (Figure 1). Thermal conditions in the 30 days prior to organism collections at each site were warmest in the north (Sawtell, 25.3°C ± 0.4°C, mean ± SD) and coolest in the south (Fortescue, 18.1°C ± 0.1°C; Table 1).

**TABLE 1:** Mean temperature (°C ± SD) at each site in the 30 days prior to collection of specimens.

Site	Temperature
Sawtell	25.3 ± 0.4
Forster	24.4 ± 0.3
Shellharbour	24.15 ± 0.3
Merimbula	23.2 ± 0.8
Mallacoota	20.6 ± 0.4
Fortescue	18.1 ± 0.3

#### 3.1.1 Photosynthesis

**FIGURE 2:**
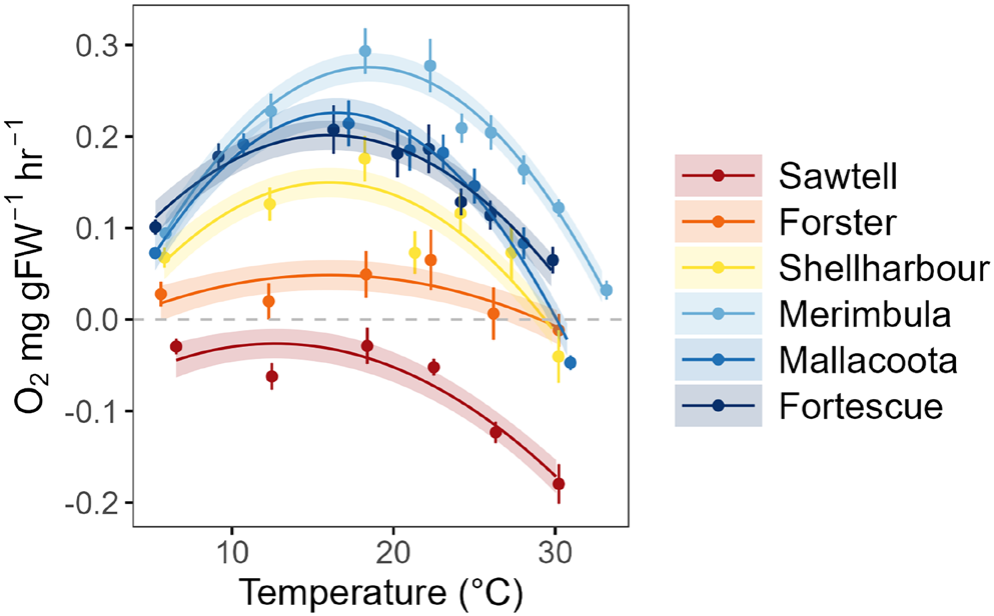
Photosynthetic rates of *Ecklonia radiata* at each site across latitude. Points show the mean of *n* = 9 replicates (±SE) and solid lines represent the model‐predicted mean (±SE in shading). Colours for each site represent a gradient from warm‐edge (red) to cool‐edge (blue) locations.

**FIGURE 3:**
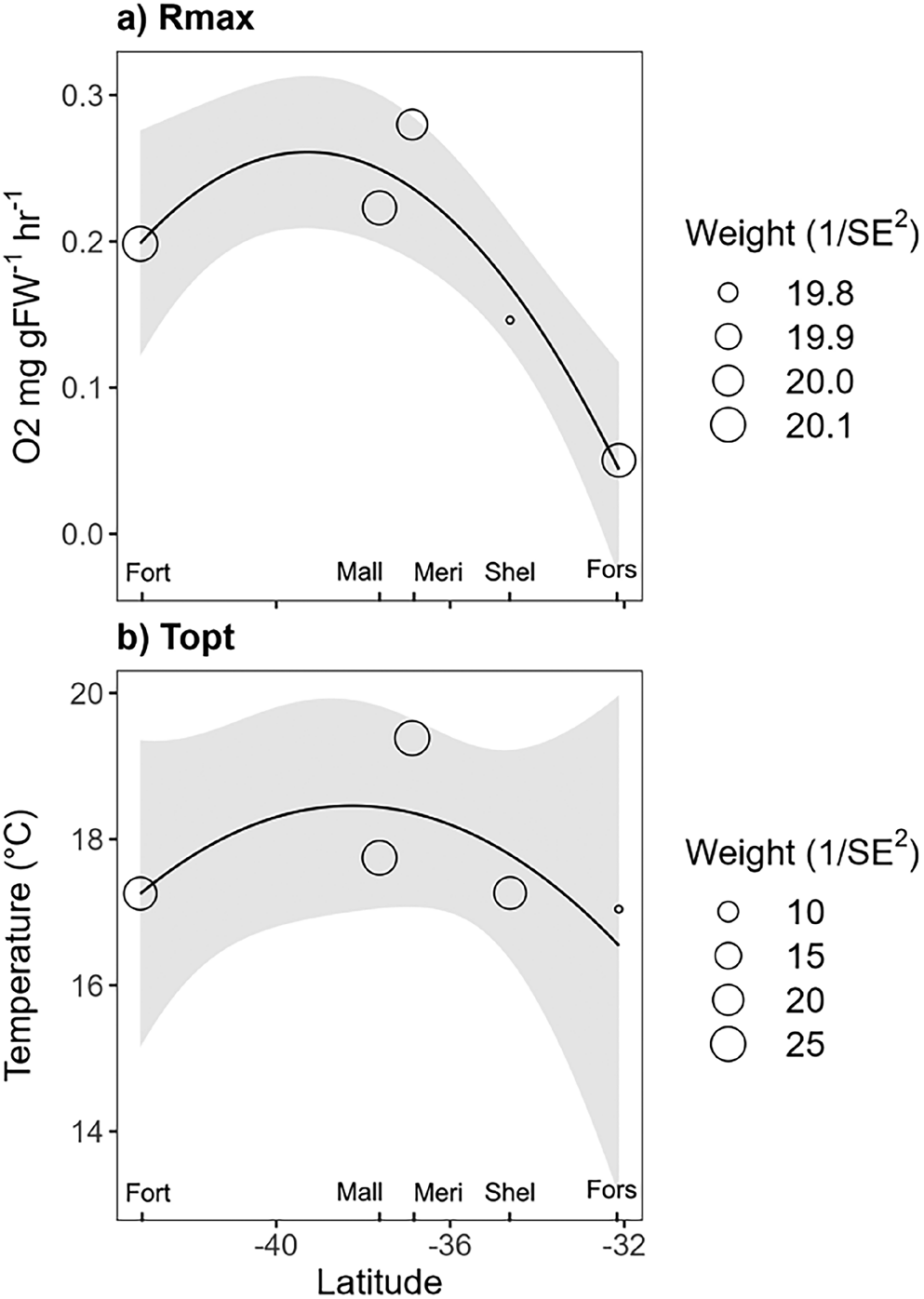
(a) Maximum rate of and (b) optimum temperature for photosynthesis of *E. radiata* at each site across latitude. Points in each panel represent the mean value with point size showing the inverse of the sampling variance: This indicates the weight given to each data point in the model, with larger sizes meaning the point was weighted more heavily (i.e., was more precise with lower standard error). $R_{\max}$ and $T_{\mathrm{opt}}$ values for the warmest site (Sawtell) are not shown because there was no net gain in $O_{2}$ observed at Sawtell and therefore it was not included in the models.

Clear patterns in the maximum rate of and optimum temperature for photosynthesis were observed across latitude in *E. radiata* (Figure 2). The maximum rate of photosynthesis showed a significant and unimodal curve across latitude (Figure 3a; Table 2). $R_{\max}$ peaked in centre‐range sites, with the highest rate observed at Merimbula (Figure 3a). A steep decline in rate was observed from centre‐range populations towards warmer waters, and at the warmest site (Sawtell), the rate of oxygen production was insufficient to record any net gain in $O_{2}$ (Figure 3a). From centre‐range to cool‐edge locations, the decline is less pronounced (Figure 3a).

**TABLE 2:** Results of the meta‐regression used to assess the trend across latitude in the maximum rate of photosynthesis and the optimum temperature for photosynthesis in *E. radiata* .

Parameter	*F*‐stat	df	*p*	AIC
*Rmax*	21.61	2	<0.000	0.35
*Topt*	1.24	2	0.54	14.46

The optimum temperature for photosynthesis ranged a mean of 15.6 ± 0.08 at Forster, to 18.4 ± 0.1 at Merimbula 3b. There was no significant change in *Topt* for photosynthesis across latitude (Figure 3b, Table 2).

#### 3.1.2 Urchin Respiration

**FIGURE 4:**
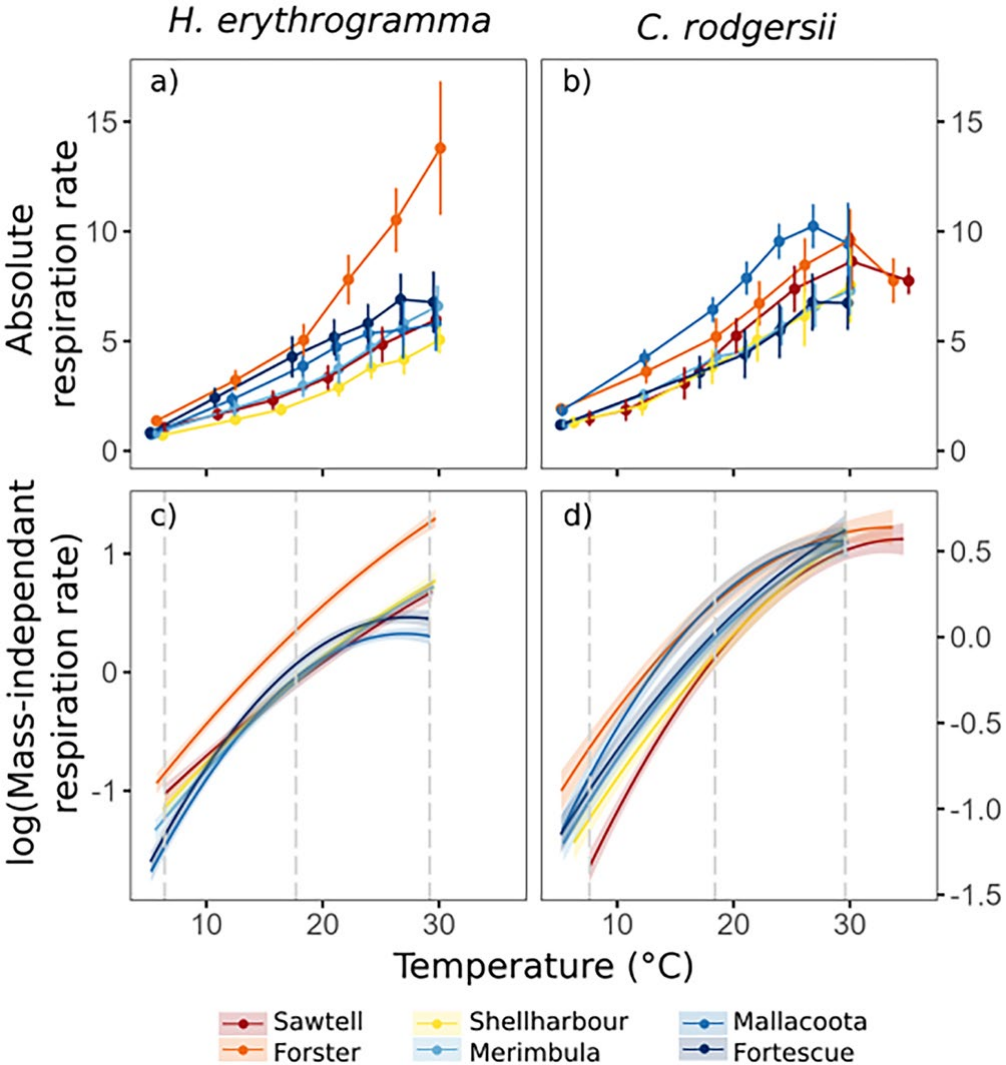
Respiration rates (mg O^2^ h^−1^) for *H. erythrogramma* (a, c), and *C. rodgersii* (b, d), at each site. Top row shows absolute values (mean of *n* = 9 replicates ± 95% CI) and the bottom row shows predicted mass‐independent rates (mean ± 95% CI).

Respiration rates of both urchin species increased with temperature up until urchin death at each site across latitude (Figure 4). For some species and site combinations (for *C. rodgersii* at Sawtell, Forster and Mallacoota and for *H. erythrogramma* at Mallacoota and Fortescue), a slight decrease in metabolic rate was observed at the highest temperature tested before urchin death (Figure 4).

*Heliocidaris erythrogramma* and *C. rodgersii* differed in their oxygen consumption rates (LMM: *p* < 0.000; Table 3). Overall, *H. erythrogramma* consumed 22.1% less oxygen per individual compared to *C. rodgersii*, with a mean of 4.2 ± 0.16 mg O^2^ h^−1^ (mean ± SE) relative to 5.3 ± 0.16 mg $O_{2}$ $h^{-1}$ (mean ± SE) for *C. rodgersii* (Table 3).

**TABLE 3:** Results of the linear mixed effects model used to compare overall respiration rates between species.

Fixed effects					
Term	Estimate	Std. error	df	*t*	Pr(>∣ *t* ∣)
(Intercept)	1.43	0.09	106	16.61	<0.000
speciesHelio	−0.25	0.08	448	−4.39	<0.000

Random effects			
Group	Name	Variance	Std. dev
Temp	(intercept)	0.45	0.67
Latitude:Replicate	(intercept)	0.05	0.22
Residual		0.04	0.21

*Note:* No. observations: 702. No. groups: Temp, 74; Latitude: Replicate, 57.

**FIGURE 5:**
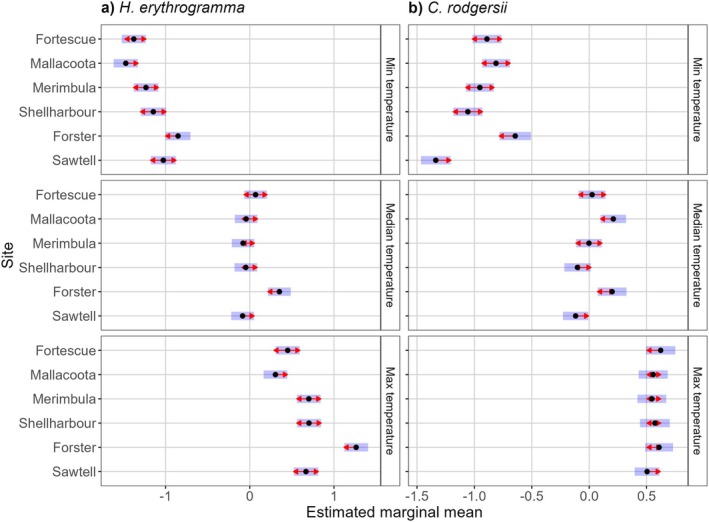
Pairwise differences in estimated marginal means for respiration rates of (a) *H. erythrogramma* and (b) *C. rodgersii* at different sites across latitude. Pairwise comparisons were carried out at the minimum, median and maximum temperature of the shared temperature range that was tested across locations. For *H. erythrogramma* this was 6.42°C, 17.7°C, 29.2°C while for *C. rodgersii* these temperatures were 7.62°C, 18.4°C, 29.6°C. Points show the estimated marginal mean, with 95% confidence intervals shown by the blue box. The red arrows show the pairwise comparison, with significant differences between groups represented by arrows that do not overlap.

For *H. erythrogramma*, different patterns in respiration were observed between different sites (Figure 4a,c). The two cool‐edge sites showed a plateau in respiration rates at the highest temperatures tested, while for central and warm‐edge populations respiration rates were still increasing at these temperatures (Figure 4c). Analysis of deviance revealed a significant interaction between temperature and site (Wald $\chi_{\left(10\right)}^{2}$ = 142.4, *p* < 0.001; Table 4). Site comparisons of estimated marginal means at 6.42°C show significantly lower respiration rates for individuals at the cool‐edge relative to those at the warm‐edge, with central populations showing respiration rates in between these (Figure 5a; Table S9). At 17.7°C respiration rates were similar at all sites except for Forster, where rates were significantly higher (Figure 5a; Table S9). At 29.2°C respiration rates at cool‐edge sites were again lower than at the warmer sites, although for Fortescue this difference was not significant (Figure 5a, Table S9).

**TABLE 4:** Results from the analysis of deviance used to test the significance of the model fixed effects for both *H. erythrogramma* and *C. rodgersii*.

Species	Fixed effect	Chisq	df	Pr(>Chisq)
*H. erythrogramma*	poly(temp, 2)	7702.14	2	0.0000
	site	53.80	5	0.0000
	poly(temp, 2):site	142.39	10	0.0000
*C. rodgersii*	poly(temp, 2)	4112.14	2	0.0000
	site	25.80	5	0.0001
	poly(temp, 2):site	65.97	10	0.0000

For *C. rodgersii*, a latitudinal pattern in thermal sensitivity is evident, with warmer sites being more sensitive (i.e., steeper respiration vs. temperature slope) than cooler sites, except for Forster which showed the shallowest slope (Figure 4b,d). Overall, there was a larger difference in respiration rates between sites at the lower test temperatures compared to higher test temperatures, and respiration rates from each site appear to converge at the highest temperatures tested (Figures 4c and 5b). Analysis of deviance demonstrates a significant interaction between temperature and site (Wald $\chi_{\left(10\right)}^{2}$ = 66.0, *p* < 0.001; Table 4). Site comparisons of estimated marginal means at 7.62°C show significantly lower respiration rates for individuals the warm‐edge (Sawtell) compared to all other sites (Figure 5b, Table S10). At 18.4°C respiration rates have begun to converge (Figure 5b, Table S10), and at 29.6°C there are no significant differences in respiration rates between any sites (Figure 5b, Table S10).

## 4 Discussion

We measured acute thermal performance of two ecologically important urchin herbivores and their macroalgal food source at multiple sites across their latitudinal distribution. Overall, we found clear differences in performance between each sea urchin and the kelp *E. radiata* . *Ecklonia radiata* demonstrated a clear latitudinal pattern in thermal response, with declining rates of photosynthesis towards warmer waters but similar optimum temperatures for photosynthesis across latitude. Similarly, *H. erythrogramma* demonstrated a latitudinal trend in respiration rates, with cool‐edge populations showing a plateau in respiration at the higher temperatures tested, while respiration rates in central and warm‐edge populations continued to increase. For *C. rodgersii*, populations at the warm range edge showed increased sensitivity to temperature change. These patterns provide novel insights into the comparative thermal performance of kelp and urchins across their entire latitudinal distribution, with implications for their responses to ocean warming.

### 4.1 Latitudinal Patterns in Performance Indicate Species Response to Warming

For *E. radiata* , we hypothesised that thermal performance would differ between sites, reflecting this species' low dispersal capacity and subsequent genetic isolation of different populations. Our data only partially supported this expectation. We recorded a significant decrease in the maximum rate of photosynthesis across latitude, although no significant change in $T_{\mathrm{opt}}$ of photosynthesis. These results reflect those recorded in a similar study on the west coast of Australia (Wernberg, de Bettignies, et al. 2016), and suggest that while performance varies across latitude, only limited physiological adjustment to local temperature regimes had occurred. This is surprising, because thermal plasticity between populations that reflects local temperature regimes is typically common in macroalgae (King et al. 2018), and has been found among gametophyte stages for *E. radiata* (Veenhof et al. 2024; Mohring et al. 2014). Work by Coleman and colleagues (Coleman et al. 2009, 2020) even suggests there may be a genetic basis to this variation, indicating populations across its range may be similarly vulnerable to warming (e.g., Bennett et al. 2015). However, regardless of whether these differences are the result of phenotypic plasticity or genetic adaptation, the short‐term nature and rapid onset of heatwaves mean that we might expect a similar reduction in performance across all populations if they are living at or above their optimum temperature. In this case, optimum temperatures for photosynthesis across all sites was substantially lower than average summer temperature across the study region. Certainly, the significantly reduced performance we recorded for *E. radiata* at the centre and warm‐edge of its range suggests that this species is approaching its thermal limit and, overall, further warming will compromise performance across its distribution.

For *H. erythrogramma*, we similarly expected to observe a difference in respiration rates between populations, due to its short larval phase and consequent genetic isolation of populations across its range. Patterns in respiration rates broadly reflected this, with rates recorded in the two cool‐edge populations showing a plateau at the higher test temperatures, while respiration rates in central and warm‐edge populations continued to increase. This indicates these populations may have a lower thermal tolerance limit relative to warmer‐water populations. Such adjustments to local temperature regimes align with other comparative studies for this species (Byrne et al. 2011; Butler et al. 2026), and may be driven by acclimation or adaptation to a distinct change in oceanographic conditions that occurs around Cape Howe, between the cool‐edge and the central and warm‐edge sites (see Figure 1; Ridgway and Dunn 2003; Middleton and Cirano 2005; Luick et al. 1994; Colgan 2016). However, how the plateau in respiration rates we measured for cool‐edge populations relates to their realised temperature thresholds remains less clear. Respiration rates typically increase until an organism's lethal limit is reached, and do not reflect the temperature at which realised thermal impacts are observed (see Section 4.4 below). Further work to understand how respiration rates relate to temperature thresholds in the wild is required to better understand the effects of warming on this species.

Despite the differences in respiration rates at higher temperatures for *H. erythrogramma*, the similarity in rates between populations across the rising phase of the thermal performance curve is surprising. This lack of differentiation may reflect that respiration rates are highly plastic and can be altered in response to acute or long‐term changes in environmental conditions (Schuster et al. 2022; Storey 1988; Van Ginneken et al. 2001; Peck et al. 2014). In fact, whether or not the slope of the temperature vs. respiration relationship is altered in a consistent way for organisms acclimated to different temperatures has long been debated (Schulte 2015). A species' response to its environment is complex, involving myriad trade‐offs. Furthermore, genetic and phenotypic influences can act in opposition across environmental gradients (Conover et al. 2009; Conover and Schultz 1995), and these complexities may reduce or prevent any observed phenotypic change between populations experiencing different environmental conditions.

For *C. rodgersii*, we expected to see no difference in thermal performance between populations, owing to this species' long larval phase and consequent high dispersal capacity, both of which are life history traits often associated with species that occupy a conserved thermal niche (Bennett et al. 2019). Our data supported this expectation, with respiration rates converging at the highest test temperatures, suggesting a conserved upper thermal limit. However, respiration rates for *C. rodgersii* also showed a latitudinal pattern in thermal sensitivity, with populations in warmer waters more sensitive to temperature change than those at the cooler sites (with the exception of Forster). Increased thermal sensitivity at warmer sites suggests *C. rodgersii* may be more vulnerable to temperature impacts in these locations. Increased sensitivity in species experiencing thermal stress has been observed in other organisms, particularly in populations towards their warm range edge (Smith et al. 2024; Shi et al. 2026). Recent work assessing grazing rates of *C. rodgersii* across their thermal distribution has also found decreased performance (lower consumption) in warmer waters (Butler et al. 2026). The observation that warming impacts are only recorded in these warm‐edge populations is still consistent with a conserved thermal niche, but indicates populations towards their warm range edge are likely approaching their thermal limit.

For both urchin species, respiration rates at Forster were higher than would be expected relative to other sites, particularly for *H. erythrogramma*. This pattern was consistent across absolute, mass‐standardised and mass‐independent respiration rates, indicating that this pattern cannot be attributed to differences in urchin body size between sites. The higher respiration rates observed at Forster may reflect a difference in conditions at this particular site. For *H. erythrogramma*, organisms from this site were only found in shallow depths (∼0–1 m) in an area that received only intermittent ocean water influx from mid to low tides. These organisms may have experienced increased thermal stress in this habitat, particularly on warm and calm days. This may have influenced their respiration rates and thermal response relative to urchins from other sites that were found slightly deeper (1–3 m) and in open‐water locations (McMahon et al. 1995).

### 4.2 Species Relative Thermal Limits

*Ecklonia radiata* and *H. erythrogramma* have a cooler thermal affinity relative to *C. rodgersii*, and we hypothesised that these species' thermal limits would reflect this difference. Our data show evidence of decreasing performance towards warmer latitudes for *E. radiata* and *C. rodgersii*, suggesting that performance of each species will follow a broadly negative trajectory under longer‐term warming. For *H. erythrogramma*, results suggest a similar pattern. Although we cannot directly compare thermal limits between photosynthetic and respiration rates, particularly considering respiration rates overestimate realised thresholds (see Section 4.4 below), the different patterns of performance, and the mechanisms by which thermal stress was evident in *E. radiata* and *C. rodgersii* indicate that *E. radiata* has a slightly lower thermal threshold compared to *C. rodgersii*. For *E. radiata* , a severe decrease in maximum rate of photosynthesis recorded from central to warm‐edge populations suggests this species experiences physiological limitation in warmer waters. Lower rates of photosynthesis in warmer waters have also been recorded on the west coast of Australia (Wernberg, de Bettignies, et al. 2016), attributed to lower concentrations of Chlorophyll *a*. Reduced concentrations of Chl*a* would lower the light‐harvesting capacity of *E. radiata* , with subsequent impacts on growth and reproduction. A reduced capacity to acquire energy may also impact its ability to cope with thermal stress. In comparison, *C. rodgersii* shows increased sensitivity to acute warming, yet no physiological evidence of longer‐term thermal stress, indicating that their thermal threshold may be relatively higher. However, despite this, the short‐term nature and rapid onset of marine heatwaves mean that we might expect reductions in performance for all species across their ranges.

Latitudinal patterns in respiration rates of *H. erythrogramma* show cool‐edge populations to have a lower temperature threshold relative to central and warm‐edge populations. This indicates potential for acclimation or adaptation to local conditions. Given that laboratory experiments carried out in warm‐edge populations show this species may be approaching its upper tolerance threshold (Byrne et al. 2011, 2009; Harianto et al. 2018; Gall et al. 2021), this suggests that populations of *H. erythrogramma* at other locations across their range may be similarly vulnerable to further warming (Bennett et al. 2015). Further research investigating the thermal tolerance of different populations of *H. erythrogramma* over their latitudinal distribution is warranted.

### 4.3 Ecological Implications

Understanding what these patterns in performance and relative thermal limits mean for the strength of the interaction between these species (i.e., grazing rates), and how this impacts temperate reef ecosystems is challenging. Previous research for these species provides some interesting context, and suggests that grazing rates may begin declining as organisms approach their upper thermal limits (Butler et al. 2026; Minuti et al. 2021). For example, Minuti et al. (2021) found that *H. erythrogramma* grazing rates did not increase alongside respiration rates when exposed to acute thermal stress, and Butler et al. (2026) compared in situ grazing rates of *C. rodgersii* and *H. erythrogramma* across the latitudinal distribution of these species in eastern Australia and found that *C. rodgersii* grazing rates peaked close to the mid‐point of their range, whereas *H. erythrogramma* grazing rates remained relatively constant throughout. While these findings cannot speak to the thermal sensitivity of grazing rates within populations, they do suggest grazing rates may decline when under thermal stress, with *H. erythrogramma* showing greater plasticity in grazing rates in response to temperature. Laboratory experiments that can compare the thermal sensitivity of grazing rates and respiration rates at multiple locations across these species range would provide further insight into how warming may influence the impact of these urchins on temperate reefs. Ultimately, considering that our results indicate that *E. radiata* has a slightly lower thermal threshold compared to *C. rodgersii*, this suggests that interaction strength may increase in regions where *E. radiata* is at or above its thermal optimum, compounding grazing impacts. For *H. erythrogramma*, results are less clear, but given this species may be approaching it's upper thermal limits across its range (Byrne et al. 2011; Butler et al. 2026; Harianto et al. 2018; Gall et al. 2021), a reduction in grazing pressure may occur.

Another consideration in understanding this interaction between sea urchins and kelps under warming is kelp palatability. Changes in the chemical composition and nutritional quality of kelp with warming are well documented, although the direction and magnitude of this are context dependent (Poore et al. 2013; Harley et al. 2012; Castro et al. 2024). For *E. radiata* , Castro et al. (2024) show that although the nutritional value and chemical defences of the kelp remained similar under warming, the microorganisms associated with tissue disease and degradation increased, leading to higher consumption by a tropical urchin herbivore. To what extent this may be true for *C. rodgersii* (an omnivore; Caley et al. (2021)) and *H. erythrogramma* (a more strict herbivore; Vanderklift and Wernberg (2010)), and how this may hold at temperatures approaching these species' thermal limits, is not known.

### 4.4 Respiration Rates as a Predictor of Species Response to Warming

**FIGURE 6:**
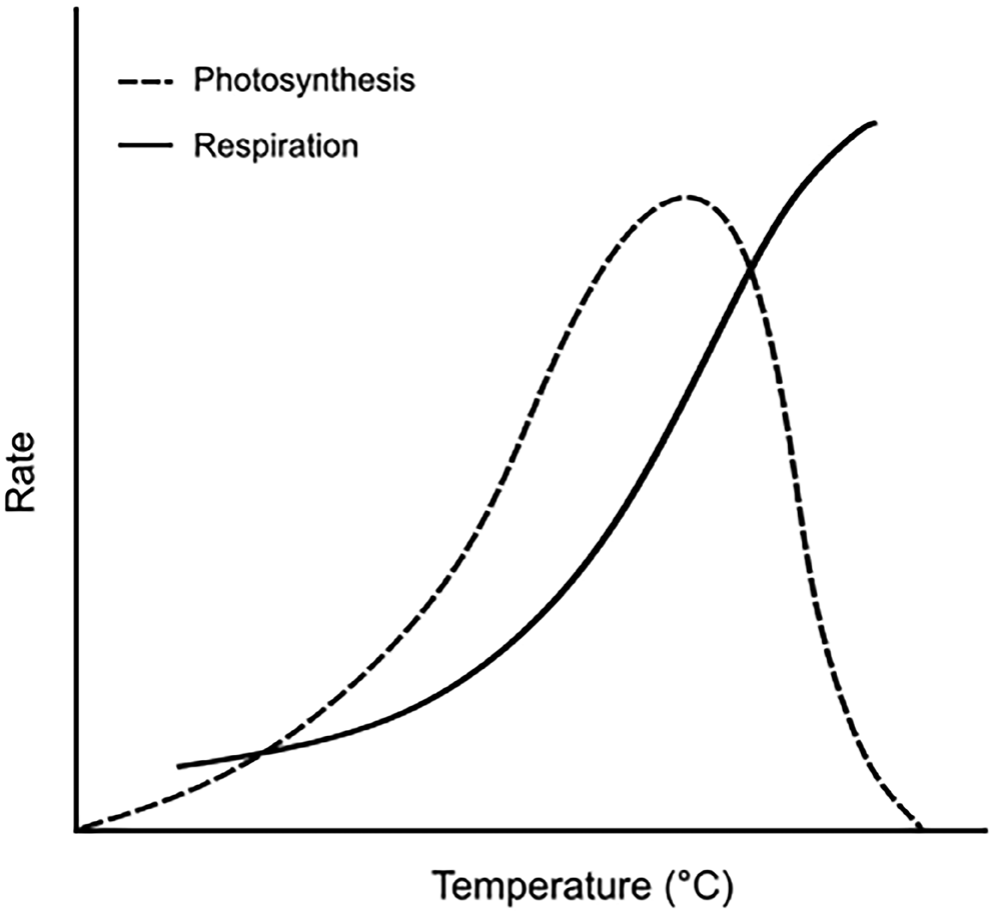
A typical thermal performance curve as observed for the different processes of photosynthesis (dashed line) and respiration (solid line). For photosynthesis (and other biochemical rates), thermal performance curves generally show a rising phase, a peak, and a falling phase. Although in theory thermal performance curves for respiration rates would follow a similar shape, in reality the downturn is difficult to measure because organisms reach their critical thermal maximum. Given the peak of a respiration thermal performance curve corresponds to a critical or lethal limit, rather than a thermal optimum, careful interpretation is needed to avoid overestimating thermal limits.

Respiration rates are a commonly used metric for assessing a species' response to warming. They are common to all living organisms, describe an organism's fundamental cost of existence, and also underpin theories such as the metabolic theory of ecology (Brown et al. 2004) and oxygen and capacity limited thermal tolerance (H. O. Pörtner 2001, 2002, 2010; Pörtner et al. 2017). However, the ecological interpretation of respiration rates in this context requires careful consideration, because the peak of a respiration‐temperature curve can substantially overestimate the organismal thermal limits, particularly in the wild. Unlike a typical thermal performance curve for metrics such as photosynthesis, growth and reproduction, which are characterised by a thermal optimum and a decline in performance thereafter, respiration rates increase until an organism's lethal limit is reached (Figure 6)—reflecting the disconnect in thermal performance at different levels of biological organisation (Rezende and Bozinovic 2019). This creates challenges for using respiration rates as a tool to predict the thermal threshold for climate change impacts, as they do not reflect the realised temperatures at which impacts occur. Yet despite this disconnect, respiration rates contribute important information to understanding the impacts of temperature. Respiration rates are useful to understand relative differences in performance between populations and species (as in this study), and the relationship between respiration and other ecologically important traits offers potential for a better understanding of community and ecosystem wide responses (Brandl et al. 2023). Respiration rates can also be used alongside other metrics of performance (e.g., growth, feeding rates, neuromuscular function, survival) to provide broader context around an organisms thermal limits (Minuti et al. 2021; Pagès et al. 2018). Critically, respiration rates provide a comparable and logistically feasible means of assessing species response across large spatial and temporal scales. Given the importance of understanding patterns and processes at scale (Brown et al. 2002), this highlights their utility in understanding temperature impacts when careful consideration and calibration is given to their interpretation.

## 5 Conclusions

Our data demonstrate that further warming or heatwave events will likely compromise the performance of two urchin species and their macroalgal food source, although these impacts may differ across warm‐edge, central and cool‐edge range positions. For *E. radiata* , performance declines may be particularly strong in central and warm‐edge locations, while for *C. rodgersii* populations may be most sensitive at their warm range edge. Results for *H. erythrogramma* indicate that this species may be adapted or acclimated to local environmental conditions, suggesting that populations across its range may be equally vulnerable to a given increase in temperature. Considering the impact of these urchins on temperate reefs in this region, via grazing of kelp species, these results are important for understanding the resilience of these ecosystems in a warmer future. By uncovering spatial variation in species response to warming, this work highlights that management and conservation strategies for these species and ecosystem may need to be tailored according to region. Further work investigating how performance relates to rates of herbivory across these species' range should be a priority.

## Author Contributions

**Claire Butler:** conceptualization (equal), formal analysis (lead), funding acquisition (supporting), methodology (equal), writing – original draft (lead). **Catriona L. Hurd:** methodology (equal), writing – review and editing (equal). **Adriana Vergés:** methodology (equal), writing – review and editing (equal). **Scott Ling:** methodology (equal), writing – review and editing (equal). **Scott Bennett:** conceptualization (equal), funding acquisition (lead), methodology (equal), writing – review and editing (equal).

## Funding

Funding for this project was provided by the Australian Research Council to Scott Bennett (DE200100900), and from the Holsworth Wildlife Research Endowment (B0028858) and the Australian Wildlife Society to Claire Butler. Catriona L. Hurd and Adriana Vergés were supported by the ARC Discovery Projects DP200101467 and DP190102030 respectively, and Scott Ling was supported by the ARC Future Fellowship FT200100949.

## Conflicts of Interest

The authors declare no conflicts of interest.

## Supporting information


**Table S1:** Mean wet weight (±SE) of urchins used in metabolic experiments at each site.
**Figure S1:** The custom‐built experimental respiratory system. Shown are (a) ten 4.5 L acrylic respiratory chambers, sitting on an aluminium frame housed within a 350 L water bath. Each chamber has internal water circulation through pumps with inlet/outlet hose inserted through the base. Oxygen sensor spots are attached to the base of the lid, and oxygen probes aligned (via screw ports) directly above (within the chimney to avoid inundation). Temperature probes are inserted into each chamber through a port in the lid. One chamber is used as a control and allows measurement of background (microorganism) respiration. The water bath contains heating and chilling elements, and water pumps to ensure homogeneous temperature throughout. (b) To measure photosynthetic rates, a frame of plastic conduit was assembled and 4 LED lights were hung above the water bath. (c) The whole set‐up was then covered in a black tarpaulin to prevent additional daylight altering these standardised conditions.
**Figure S2:** Leave‐one‐out model diagnostics for the maximum rate of photosynthesis of across latitude. Plots show different computed influence metrics for each data point on the model fit. Thresholds specific to each influence metric are shown by blue dashed lines, and points outside these thresholds (i.e., those with high influence) are shown in red. An explanation of each of the influence metrics can be viewed at https://wviechtb.github.io/mtafor/reference/influence.rma.uni.html.
**Table S2:** Summary statistics from the meta‐regression used to assess the trend in the maximum rate of, and optimum temperature for photosynthesis of across latitude, but excluding Merimbula. Sensitivity diagnostics identified this site as exerting a strong influence on the model results. Removing this site did not change the statistical significance of any model parameters.
**Figure S3:** Figures showing the trend in (a) the maximum rate of photosynthesis and (b) the optimum temperature for photosynthesis of across latitude, but excluding Merimbula from the model fitting. This site showed high influence on the model results. However, excluding Merimbula did not change the latitudinal pattern in R_*max*_ or T_*opt*_. Points in each panel represent the mean value of the given parameter with point size showing the inverse of the sampling variance. This indicates the weight given to each data point in the model, with larger sizes meaning the point was weighted more heavily (i.e., was more precise with lower standard error). In addition to excluding the parameter estimate from the highly influential site, the parameter values for the warmest site (Sawtell) were also not included in model fitting because there was no net gain in O2 observed at this site.
**Figure S4:** Leave‐one‐out model diagnostics for the optimum temperature for photosynthesis of across latitude. Plots show different computed influence metrics for each data point on the model fit. Thresholds specific to each influence metric are shown by blue dashed lines, and points outside of these thresholds (i.e., those with high influence) are shown in red. An explanation of each of the influence metrics can be viewed at https://wviechtb.github.io/metafor/reference/influence.rma.uni.html
**Figure S5:** Mass‐standardised respiration rates (mg O_2_ h^−1^) for (a) and (b), at each site across latitude.
**Table S3:** Results from the analysis of deviance for used to test the significance of the model fixed effects for mass‐standardised respiration rates of.
**Table S4:** A matrix showing the pairwise differences in estimated marginal means for mass‐standardised respiration rates of at different sites across latitude. The matrix shows the estimated marginal means along the diagonal, the *p*‐value in the upper triangle, and the differences between the estimated marginal means the lower triangle. Pairwise comparisons were carried out at the minimum (7:62°C), median (18:4°C) and maximum (29:6°C) temperature of the shared temperature range that was tested across locations.
**Table S5:** Results from the analysis of deviance for used to test the significance of the model fixed effects for mass‐standardised respiration rates of.
**Table S6:** A matrix showing the pairwise differences in estimated marginal means for mass‐standardised respiration rates of at different sites across latitude. The matrix shows the estimated marginal means along the diagonal, the *p*‐value in the upper triangle, and the differences between the estimated marginal means the lower triangle. Pairwise comparisons were carried out at the minimum (7:62°C), median (18:4°C) and maximum (29:6°C) temperature of the shared temperature range that was tested across locations.
**Figure S6:** Pairwise differences in estimated marginal means for mass‐standardised respiration rates of (a) and (b) at different sites across latitude. Pairwise comparisons were carried out at the minimum, median and maximum temperature of the shared temperature range that was tested across locations. For this was 6:42°C, 17:7°C, 29:2°C while for these temperatures were 7:62°C, 18:4°C, 29:6°C. Points show the estimated marginal mean, with 95% confidence intervals shown by the blue box. The red arrows show the pairwise comparison, with significant differences between groups represented by arrows that do not overlap.
**Table S7:** Treatment temperatures (°C) for each species at each site. Target temperatures were varied between locations and species in order to best capture the peak of the thermal performance curve.
**Table S8:** The dates on which the metabolic experiments were carried out for each species and location.
**Table S9:** A matrix showing the pairwise differences in estimated marginal means for mass‐independent respiration rates of at different sites across latitude. The matrix shows the estimated marginal means along the diagonal, the *p*‐value in the upper triangle, and the differences between the estimated marginal means the lower triangle. Pairwise comparisons were carried out at the minimum (7:62°C), median (18:4°C) and maximum (29:6°C) temperature of the shared temperature range that was tested across locations.
**Table S10:** A matrix showing the pairwise differences in estimated marginal means for mass‐independent respiration rates of at different sites across latitude. The matrix shows the estimated marginal means along the diagonal, the *p*‐value in the upper triangle, and the differences between the estimated marginal means the lower triangle. Pairwise comparisons were carried out at the minimum (7:62°C), median (18:4°C) and maximum (29:6°C) temperature of the shared temperature range that was tested across locations.

## Data Availability

The dataset and code created for this manuscript will be made publicly available at https://github.com/cbutler2/metabolics‐latitude‐urchinkelp upon publication. For review purposes, data and scripts have been uploaded in the file upload section under for review but not for publication.

## References

[ece373910-bib-0001] Angilletta, M. J. , P. H. Niewiarowski , and C. A. Navas . 2002. “The Evolution of Thermal Physiology in Ectotherms.” Journal of Thermal Biology 27: 249–268. 10.1016/S0306-4565(01)00094-8.

[ece373910-bib-0002] Bennett, A. F. 1987. “Interindividual Variability: An Under‐Utilized Resource.” In New Directions in Ecological Physiology, edited by M. Feder , A. Bennett , W. Burggren , and R. Huey . Cambridge University Press.

[ece373910-bib-0003] Bennett, S. , C. M. Duarte , N. Marba , and T. Wernberg . 2019. “Integrating Within‐Species Variation in Thermal Physiology Into Climate Change Ecology.” Philosophical Transactions of the Royal Society, B: Biological Sciences 347: 10. 10.1098/rstb.2018.0550. PMC6606463 31203756

[ece373910-bib-0004] Bennett, S. , R. Vaquer‐Sunyer , G. Jordá , M. Forteza , G. Roca , and N. Marbà . 2022. “Thermal Performance of Seaweeds and Seagrasses Across a Regional Climate Gradient.” Frontiers in Marine Science 9: 733315. 10.3389/fmars.2022.733315/full.

[ece373910-bib-0005] Bennett, S. , T. Wernberg , B. Arackal Joy , T. de Bettignies , and A. H. Campbell . 2015. “Central and Rear‐Edge Populations Can Be Equally Vulnerable to Warming.” Nature Communications 6: 10280. 10.1038/ncomms10280. PMC4703895 26691184

[ece373910-bib-0006] Bennett, S. , T. Wernberg , S. D. Connell , A. J. Hobday , C. R. Johnson , and E. S. Poloczanska . 2016. “The ‘Great Southern Reef’: Social, Ecological and Economic Value of Australia's Neglected Kelp Forests.” Marine and Freshwater Research 67: 47–56. 10.1071/MF15232.

[ece373910-bib-0007] Brandl, S. J. , J. S. Lefcheck , A. E. Bates , D. B. Rasher , and T. Norin . 2023. “Can Metabolic Traits Explain Animal Community Assembly and Functioning?” Biological Reviews 98: 1–18. 10.1111/brv.12892. 36054431

[ece373910-bib-0008] Brown, J. H. , J. F. Gillooly , A. P. Allen , V. M. Savage , and G. B. West . 2004. “Toward a Metabolic Therory of Ecology.” Ecology 85: 1771–1789. 10.1890/03-9000.

[ece373910-bib-0009] Brown, J. H. , J. F. Gillooly , and G. B. West . 2002. “The Next Step in Macroecology: From General Empirical Patterns to Universal Ecological Laws.” In Macroecology: Concepts and Consequences, 64–84. Blackwell Science.

[ece373910-bib-0010] Butler, C. , Y. Wang , C. J. Brown , et al. 2026. “Contrasting Patterns in Kelp Consumption Across Latitude by Two Barren Forming Sea Urchin Species.” Scientific Reports 16: 9069. 10.1038/s41598-025-33714-z. 41690941 PMC12992891

[ece373910-bib-0011] Byrne, M. , M. Ho , P. Selvakumaraswamy , H. D. Nguyen , S. A. Dworjanyn , and A. R. Davis . 2009. “Temperature, but Not pH, Compromises Sea Urchin Fertilization and Early Development Under Near‐Future Climate Change Scenarios.” Proceedings of the Royal Society B: Biological Sciences 276: 1883–1888. 10.1098/rspb.2008.1935. PMC2674501 19324767

[ece373910-bib-0012] Byrne, M. , P. Selvakumaraswamy , M. Ho , E. Woolsey , and H. Nguyen . 2011. “Sea Urchin Development in a Global Change Hotspot, Potential for Southerly Migration of Thermotolerant Propagules.” Deep Sea Research Part II: Topical Studies in Oceanography 58: 712–719. 10.1016/j.dsr2.2010.06.010.

[ece373910-bib-0013] Caley, A. , A. Vergés , M. Byrne , and E. Marzinelli . 2021. “Urchins Roe Quality, Morphology and Diet Vary With Depth and Distance From Kelp.” Ph.D. thesis, University of New South Wales, Sydney.

[ece373910-bib-0014] Castro, L. C. , A. Vergés , S. C. Straub , et al. 2024. “Effect of Marine Heatwaves and Warming on Kelp Microbiota Influence Trophic Interactions.” Molecular Ecology 33: e17267. 10.1111/mec.17267. 38230446

[ece373910-bib-0015] Clarke, A. , and N. M. Johnston . 1999. “Scaling of Metabolic Rate With Body Mass and Temperature in Teleost Fish.” Journal of Animal Ecology 68: 893–905. 10.1046/j.1365-2656.1999.00337.x. 20180875

[ece373910-bib-0016] Coleman, M. A. , B. M. Gillanders , and S. D. Connell . 2009. “Dispersal and Gene Flow in the Habitat‐Forming Kelp, *Ecklonia radiata*: Relative Degrees of Isolation Across an East ‐ West Coastline.” Marine and Freshwater Research 60: 802–809. 10.1071/MF08268.

[ece373910-bib-0017] Coleman, M. A. , A. J. P. Minne , S. Vranken , and T. Wernberg . 2020. “Genetic Tropicalisation Following a Marine Heatwave.” Scientific Reports 10: 12726. 10.1038/s41598-020-69665-w. 32728196 PMC7391769

[ece373910-bib-0018] Colgan, D. J. 2016. “Marine and Estuarine Phylogeography of the Coasts of South‐Eastern Australia.” Marine and Freshwater Research 67: 1597–1610. 10.1071/MF15106.

[ece373910-bib-0019] Conover, D. O. , T. A. Duffy , and L. A. Hice . 2009. “The Covariance Between Genetic and Environmental Influences Across Ecological Gradients: Reassessing the Evolutionary Significance of Countergradient and Cogradient Variation.” Annals of the New York Academy of Sciences 1168: 100–129. 10.1111/j.1749-6632.2009.04575.x. 19566705

[ece373910-bib-0020] Conover, D. O. , and E. T. Schultz . 1995. “Phenotypic Similarity and the Evolutionary Significance of Countergradient Variation.” Trends in Ecology & Evolution 10: 248–252. 10.1016/S0169-5347(00)89081-3. 21237029

[ece373910-bib-0021] Dahlke, F. T. , S. Wohlrab , M. Butzin , and H.‐O. Pörtner . 2020. “Thermal Bottlenecks in the Life Cycle Define Climate Vulnerability of Fish.” Science 369: 65–70. 10.1126/science.aaz3658. 32631888

[ece373910-bib-0022] DeLong, J. P. , G. Bachman , J. P. Gibert , et al. 2018. “Habitat, Latitude and Body Mass Influence the Temperature Dependence of Metabolic Rate.” Biology Letters 14: 20180442. 10.1098/rsbl.2018.0442. 30158142 PMC6127111

[ece373910-bib-0023] Dunphy, B. J. , N. L. C. Ragg , and M. G. Collings . 2012. “Latitudinal Comparison of Thermotolerance and HSP70 Production in F2 Larvae of the Greenshell Mussel (*Perna canaliculus*).” Journal of Experimental Biology 216, no. 7: 1202–1209. 10.1242/jeb.076729. 23239885

[ece373910-bib-0024] Gaitán‐Espitia, J. D. , L. D. Bacigalupe , T. Opitz , N. A. Lagos , T. Timmermann , and M. A. Lardies . 2014. “Geographic Variation in Thermal Physiological Performance of the Intertidal Crab *Petrolisthes violaceus* Along a Latitudinal Gradient.” Journal of Experimental Biology 217, no. 24: 4379–4386. 10.1242/jeb.108217. 25394627

[ece373910-bib-0025] Gall, M. L. , S. P. Holmes , H. Campbell , and M. Byrne . 2021. “Effects of Marine Heatwave Conditions Across the Metamorphic Transition to the Juvenile Sea Urchin (Heliocidaris Erythrogramma).” Marine Pollution Bulletin 163: 111914. 10.1016/j.marpolbul.2020.111914. 33385800

[ece373910-bib-0026] Gardiner, N. M. , P. L. Munday , and G. E. Nilsson . 2010. “Counter‐Gradient Variation in Respiratory Performance of Coral Reef Fishes at Elevated Temperatures.” PLoS One 5: e13299. 10.1371/journal.pone.0013299. 20949020 PMC2952621

[ece373910-bib-0027] Giese, A. C. , A. Farmanfarmaian , and P. Doezema . 1966. “Respiration During the Reproductive Cycle in the Sea Urchin, *Strongylocentrotus purpuratus* .” Biological Bulletin 130: 192–201. 10.2307/1539696. 5934648

[ece373910-bib-0028] Harianto, J. , H. D. Nguyen , S. P. Holmes , and M. Byrne . 2018. “The Effect of Warming on Mortality, Metabolic Rate, Heat‐Shock Protein Response and Gonad Growth in Thermally Acclimated Sea Urchins (Heliocidaris Erythrogramma).” Marine Biology 165: 96. 10.1007/s00227-018-3353-8.

[ece373910-bib-0029] Harley, C. D. G. , K. M. Anderson , K. W. Demes , et al. 2012. “Effects of Climate Change on Global Seaweed Communities.” Journal of Phycology 48: 1064–1078. 10.1111/j.1529-8817.2012.01224.x. 27011268

[ece373910-bib-0030] Healy, T. M. , and P. M. Schulte . 2012. “Thermal Acclimation Is Not Necessary to Maintain a Wide Thermal Breadth of Aerobic Scope in the Common Killifish ( *Fundulus heteroclitus* ).” Physiological and Biochemical Zoology 85: 107–119. 10.1086/664584. 22418704

[ece373910-bib-0031] Huggett, M. J. , C. K. King , J. E. Williamson , and P. D. Steinberg . 2005. “Larval Development and Metamorphosis of the Australian Diadematid Sea Urchin *Centrostephanus rodgersii* .” Invertebrate Reproduction & Development 47: 197–204. 10.1080/07924259.2005.9652160.

[ece373910-bib-0032] Hurd, C. L. , P. J. Harrison , K. Bischof , and C. S. Lobban . 2014. “Light and Photosynthesis.” In Seaweed Ecology and Physiology, 2nd ed. Cambridge University Press.

[ece373910-bib-0033] Keesing, J. K. 2020. “Heliocidaris Erythrogramma.” In Sea Urchins: Biology and Ecology, vol. 43, 4th ed., 537–552. Elsevier.

[ece373910-bib-0034] Kerry, C. , and M. Roughan . 2020. “Downstream Evolution of the East Australian Current System: Mean Flow, Seasonal, and Intra‐Annual Variability.” Journal of Geophysical Research: Oceans 125: e2019JC015227. 10.1029/2019JC015227.

[ece373910-bib-0035] King, N. G. , N. J. McKeown , D. A. Smale , and P. J. Moore . 2018. “The Importance of Phenotypic Plasticity and Local Adaptation in Driving Intraspecific Variability in Thermal Niches of Marine Macrophytes.” Ecography 41: 1469–1484. 10.1111/ecog.03186.

[ece373910-bib-0036] Kirkman, H. 1981. “The First Year in the Life History and the Survival of the Juvenile Marine Macrophyte, *Ecklonia radiata* .” Journal of Experimental Marine Biology and Ecology 55: 243–254.

[ece373910-bib-0037] Kordas, R. L. , C. D. Harley , and M. I. O'Connor . 2011. “Community Ecology in a Warming World: The Influence of Temperature on Interspecific Interactions in Marine Systems.” Journal of Experimental Marine Biology and Ecology 400: 218–226. 10.1016/j.jembe.2011.02.029.

[ece373910-bib-0038] Lemoine, N. P. , and D. E. Burkepile . 2012. “Temperature‐Induced Mismatches Between Consumption and Metabolism Reduce Consumer Fitness.” Ecology 93: 2483–2489. 10.1890/12-0375.1. 23236919

[ece373910-bib-0039] Ling, S. D. 2008. “Range Expansion of a Habitat‐Modifying Species Leads to Loss of Taxonomic Diversity: A New and Impoverished Reef State.” Oecologia 156: 883–894. 10.1007/s00442-008-1043-9. 18481099

[ece373910-bib-0040] Ling, S. D. , C. R. Johnson , S. Frusher , and C. K. King . 2008. “Reproductive Potential of a Marine Ecosystem Engineer at the Edge of a Newly Expanded Range.” Global Change Biology 14: 907–915. 10.1111/j.1365-2486.2008.01543.x.

[ece373910-bib-0041] Ling, S. D. , and J. P. Keane . 2024. “Climate‐Driven Invasion and Incipient Warnings of Kelp Ecosystem Collapse.” Nature Communications 15: 400. 10.1038/s41467-023-44543-x. PMC10776680 38195631

[ece373910-bib-0042] Ling, S. D. , R. E. Scheibling , A. Rassweiler , et al. 2015. “Global Regime Shift Dynamics of Catastrophic Sea Urchin Overgrazing.” Philosophical Transactions of the Royal Society, B: Biological Sciences 370: 20130269. 10.1098/rstb.2013.0269.

[ece373910-bib-0043] Luick, J. L. , R. Ka¨se , and M. Tomczak . 1994. “On the Formation and Spreading of the Bass Strait Cascade.” Continental Shelf Research 14: 385–399. 10.1016/0278-4343(94)90025-6.

[ece373910-bib-0044] McMahon, R. F. , W. D. Russell‐Hunter , and D. W. Aldridge . 1995. “Lack of Metabolic Temperature Compensation in the Intertidal Gastropods, *Littorina saxatilis* (Olivi) and *L. obtusata* (L.).” Hydrobiologia 309: 89–100.

[ece373910-bib-0045] McMillan, W. O. , R. A. Raff , and S. R. Palumbi . 1992. “Population Genetic Consequences of Developmental Evolution in Sea Urchins (Genus Heliocidaris).” Evolution 46: 1299–1312. 10.1111/j.1558-5646.1992.tb01125.x. 28568989

[ece373910-bib-0046] Middleton, J. F. , and M. Cirano . 2005. “Wintertime Circulation Off Southeast Australia: Strong Forcing by the East Australian Current.” Journal of Geophysical Research: Oceans 110: 2004JC002855. 10.1029/2004JC002855.

[ece373910-bib-0047] Minuti, J. J. , M. Byrne , D. A. Hemraj , and B. D. Russell . 2021. “Capacity of an Ecologically Key Urchin to Recover From Extreme Events: Physiological Impacts of Heatwaves and the Road to Recovery.” Science of the Total Environment 785: 147281. 10.1016/j.scitotenv.2021.147281. 33933766

[ece373910-bib-0048] Mohring, M. , T. Wernberg , J. Wright , S. Connell , and B. Russell . 2014. “Biogeographic Variation in Temperature Drives Performance of Kelp Gametophytes During Warming.” Marine Ecology Progress Series 513: 85–96. 10.3354/meps10916.

[ece373910-bib-0049] Mos, B. , M. Byrne , and S. A. Dworjanyn . 2020. “Effects of Low and High pH on Sea Urchin Settlement, Implications for the Use of Alkali to Counter the Impacts of Acidification.” Aquaculture 528: 735618. 10.1016/j.aquaculture.2020.735618.

[ece373910-bib-0050] Norin, T. , H. Malte , and T. D. Clark . 2013. “Aerobic Scope Does Not Predict the Performance of a Tropical Eurythermal Fish at Elevated Temperatures.” Journal of Experimental Biology 217, no. 2: 244–251. 10.1242/jeb.089755. 24115064

[ece373910-bib-0051] Pagès, J. F. , T. M. Smith , F. Tomas , et al. 2018. “Contrasting Effects of Ocean Warming on Different Components of Plant‐Herbivore Interactions.” Marine Pollution Bulletin 134: 55–65. 10.1016/j.marpolbul.2017.10.036. 29074253

[ece373910-bib-0052] Peck, L. S. , S. A. Morley , J. Richard , and M. S. Clark . 2014. “Acclimation and Thermal Tolerance in Antarctic Marine Ectotherms.” Journal of Experimental Biology 217: 16–22. 10.1242/jeb.089946. 24353200

[ece373910-bib-0053] Pecl, G. T. , M. B. Araújo , J. D. Bell , et al. 2017. “Biodiversity Redistribution Under Climate Change: Impacts on Ecosystems and Human Well‐Being.” Science 355: eaai9214. 10.1126/science.aai9214. 28360268

[ece373910-bib-0054] Poloczanska, E. S. , C. J. Brown , W. J. Sydeman , et al. 2013. “Global Imprint of Climate Change on Marine Life.” Nature Climate Change 3: 919–925. 10.1038/nclimate1958.

[ece373910-bib-0055] Poore, A. G. B. , A. H. Campbell , R. A. Coleman , et al. 2012. “Global Patterns in the Impact of Marine Herbivores on Benthic Primary Producers.” Ecology Letters 15: 912–922. 10.1111/j.1461-0248.2012.01804.x. 22639820

[ece373910-bib-0056] Poore, A. G. B. , A. Graba‐Landry , M. Favret , H. Sheppard Brennand , M. Byrne , and S. A. Dworjanyn . 2013. “Direct and Indirect Effects of Ocean Acidification and Warming on a Marine Plant–Herbivore Interaction.” Oecologia 173: 1113–1124. 10.1007/s00442-013-2683-y. 23673470

[ece373910-bib-0058] Pörtner, H. O. 2001. “Climate Change and Temperature‐Dependent Biogeography: Oxygen Limitation of Thermal Tolerance in Animals.” Naturwissenschaften 88: 137–146. 10.1007/s001140100216. 11480701

[ece373910-bib-0057] Pörtner, H. O. 2002. “Climate Variations and the Physiological Basis of Temperature Dependent Biogeography: Systemic to Molecular Hierarchy of Thermal Tolerance in Animals.” Comparative Biochemistry and Physiology Part A: Molecular & Integrative Physiology 132: 739–761. 10.1016/s1095-6433(02)00045-4 12095860

[ece373910-bib-0059] Pörtner, H. O. 2010. “Oxygen‐ and Capacity‐Limitation of Thermal Tolerance: A Matrix for Integrating Climate‐Related Stressor Effects in Marine Ecosystems.” Journal of Experimental Biology 213: 881–893. 10.1242/jeb.037523. 20190113

[ece373910-bib-0060] Pörtner, H. O. , C. Bock , and F. C. Mark . 2017. “Oxygen‐ and Capacity‐Limited Thermal Tolerance: Bridging Ecology and Physiology.” Journal of Experimental Biology 220: 2685–2696. 10.1242/jeb.134585. 28768746

[ece373910-bib-0061] Rezende, E. L. , and F. Bozinovic . 2019. “Thermal Performance Across Levels of Biological Organization.” Philosophical Transactions of the Royal Society, B: Biological Sciences 374: 10. 10.1098/rstb.2018.0549. PMC6606466 31203764

[ece373910-bib-0062] Ridgway, K. , and J. Dunn . 2003. “Mesoscale Structure of the Mean East Australian Current System and Its Relationship With Topography.” Progress in Oceanography 56: 189–222. 10.1016/S0079-6611(03)00004-1.

[ece373910-bib-0063] Ridgway, K. , and S. Ling . 2023. “Three Decades of Variability and Warming of Nearshore Waters Around Tasmania.” Progress in Oceanography 215: 103046. 10.1016/j.pocean.2023.103046.

[ece373910-bib-0064] Scholander, P. F. , W. Flagg , V. Walters , and L. Irving . 1953. “Climatic Adaptation in Arctic and Tropical Poikilotherms.” Physiological Zoology 26: 67–92. 10.1086/physzool.26.1.30152151.

[ece373910-bib-0065] Schulte, P. M. 2015. “The Effects of Temperature on Aerobic Metabolism: Towards a Mechanistic Understanding of the Responses of Ectotherms to a Changing Environment.” Journal of Experimental Biology 218: 1856–1866. 10.1242/jeb.118851. 26085663

[ece373910-bib-0066] Schulte, P. M. , T. M. Healy , and N. A. Fangue . 2011. “Thermal Performance Curves, Phenotypic Plasticity, and the Time Scales of Temperature Exposure.” Integrative and Comparative Biology 51: 691–702. 10.1093/icb/icr097. 21841184

[ece373910-bib-0067] Schuster, J. M. , and A. E. Bates . 2023. “The Role of Kelp Availability and Quality on the Energetic State and Thermal Tolerance of Sea Urchin and Gastropod Grazers.” Journal of Experimental Marine Biology and Ecology 569: 151947. 10.1016/j.jembe.2023.151947.

[ece373910-bib-0068] Schuster, J. M. , A. Kurt Gamperl , P. Gagnon , and A. E. Bates . 2022. “Distinct Realized Physiologies in Green Sea Urchin ( *Strongylocentrotus droebachiensis* ) Populations From Barren and Kelp Habitats.” Facets 7: 822–842. 10.1139/facets-2021-0125.

[ece373910-bib-0069] Shi, J. , S. Bennett , J. B. Kajtar , et al. 2026. “Warm Edge Kelp Populations Show Elevated Volatility to Marine Heatwaves.” Ecology Letters 29: e70307. 10.1111/ele.70307. 41574748 PMC12828870

[ece373910-bib-0070] Smith, K. E. , M. Aubin , M. T. Burrows , et al. 2024. “Global Impacts of Marine Heatwaves on Coastal Foundation Species.” Nature Communications 15: 5052. 10.1038/s41467-024-49307-9. PMC11176324 38871692

[ece373910-bib-0071] Spillman, C. M. , G. A. Smith , A. J. Hobday , and J. R. Hartog . 2021. “Onset and Decline Rates of Marine Heatwaves: Global Trends, Seasonal Forecasts and Marine Management.” Frontiers in Climate 3: 801217. 10.3389/fclim.2021.801217.

[ece373910-bib-0072] Stillman, J. H. 2004. “A Comparative Analysis of Plasticity of Thermal Limits in Porcelain Crabs Across Latitudinal and Intertidal Zone Clines.” International Congress Series 1275: 267–274. 10.1016/j.ics.2004.09.034.

[ece373910-bib-0073] Storey, K. B. 1988. “Suspended Animation: The Molecular Basis of Metabolic Depression.” Canadian Journal of Zoology 66: 124–132. 10.1139/z88-016.

[ece373910-bib-0074] Van Ginneken, V. J. , M. Onderwater , O. L. Olivar , and G. E. Van Den Thillart . 2001. “Metabolic Depression and Investigation of Glucose/Ethanol Conversion in the European Eel ( *Anguilla anguilla* Linnaeus 1758) During Anaerobiosis.” Thermochimica Acta 373: 23–30. 10.1016/S0040-6031(01)00463-4.

[ece373910-bib-0075] Vanderklift, M. , and T. Wernberg . 2010. “Stable Isotopes Reveal a Consistent Consumer–Diet Relationship Across Hundreds of Kilometres.” Marine Ecology Progress Series 403: 53–61. 10.3354/meps08484.

[ece373910-bib-0076] Veenhof, R. J. , C. Champion , S. A. Dworjanyn , J. Schwoerbel , W. Visch , and M. A. Coleman . 2024. “Projecting Kelp (*Ecklonia radiata*) Gametophyte Thermal Adaptation and Persistence Under Climate Change.” Annals of Botany 133: 153–168. 10.1093/aob/mcad132. 37665952 PMC10921825

[ece373910-bib-0077] Vergés, A. , C. Doropoulos , R. Czarnik , K. McMahon , N. Llonch , and A. G. B. Poore . 2018. “Latitudinal Variation in Seagrass Herbivory: Global Patterns and Explanatory Mechanisms.” Global Ecology and Biogeography 27: 1068–1079. 10.1111/geb.12767.

[ece373910-bib-0078] Vergés, A. , P. D. Steinberg , M. E. Hay , et al. 2014. “The Tropicalization of Temperate Marine Ecosystems: Climate‐Mediated Changes in Herbivory and Community Phase Shifts.” Proceedings of the Royal Society B: Biological Sciences 281: 20140846. 10.1098/rspb.2014.0846. PMC4100510 25009065

[ece373910-bib-0079] Vernberg, F. J. 1959. “Studies on the Physiological Variation Between Tropical and Temperate Zone Fiddler Crabs of the Genus UCA. Ii. Oxygen Consumption of Whole Organisms.” Biological Bulletin 117: 163–184. 10.2307/1539048.

[ece373910-bib-0080] Wernberg, T. , S. Bennett , R. C. Babcock , et al. 2016. “Climate‐Driven Regime Shift of a Temperate Marine Ecosystem.” Science 353: 169–172. 10.1126/science.aad8745. 27387951

[ece373910-bib-0081] Wernberg, T. , T. de Bettignies , B. A. Joy , and P. M. Finnegan . 2016. “Physiological Responses of Habitat‐Forming Seaweeds to Increasing Temperatures.” Limnology and Oceanography 61: 2180–2190. 10.1002/lno.10362.

[ece373910-bib-0082] Wernberg, T. , M. S. Thomsen , F. Tuya , G. A. Kendrick , P. A. Staehr , and B. D. Toohey . 2010. “Decreasing Resilience of Kelp Beds Along a Latitudinal Temperature Gradient: Potential Implications for a Warmer Future: Climate and Resilience of Kelp Beds.” Ecology Letters 13: 685–694. 10.1111/j.1461-0248.2010.01466.x. 20412279

[ece373910-bib-0083] Williams, D. , and D. Anderson . 1975. “The Reproductive System, Embryonic Development, Larval Development and Metamorphosis of the Sea Urchin Heliocidaris Erythrogramma (Val.) (Echinoidea: Echinometridae).” Australian Journal of Zoology 23: 371–403. 10.1071/ZO9750371.

