# Supplementary material for: Comparative Thermal Performance of Kelp and Herbivores Across a Latitudinal Gradient in Ocean Temperature
Source: Ecol Evol. 2026 Jul 6;16(7):e73910. doi: 10.1002/ece3.73910 (PMC13337321; doi:10.1002/ece3.73910)
Supplement: Supplementary file 1 — Table S1: Mean wet weight (±SE) of urchins used in metabolic experiments at each site. Figure S1: The custom‐built experimental respiratory system. Shown are (a) ten 4.5 L acrylic respiratory chambers, sitting on an aluminium frame housed within a 350 L water bath. Each chamber has internal water circulation through pumps with inlet/outlet hose inserted through the base. Oxygen sensor spots are attached to the base of the lid, and oxygen probes aligned (via screw ports) directly above (within the chimney to avoid inundation). Temperature probes are inserted into each chamber through a port in the lid. One chamber is used as a control and allows measurement of background (microorganism) respiration. The water bath contains heating and chilling elements, and water pumps to ensure homogeneous temperature throughout. (b) To measure photosynthetic rates, a frame of plastic conduit was assembled and 4 LED lights were hung above the water bath. (c) The whole set‐up was then covered in a black tarpaulin to prevent additional daylight altering these standardised conditions. Figure S2: Leave‐one‐out model diagnostics for the maximum rate of photosynthesis of across latitude. Plots show different computed influence metrics for each data point on the model fit. Thresholds specific to each influence metric are shown by blue dashed lines, and points outside these thresholds (i.e., those with high influence) are shown in red. An explanation of each of the influence metrics can be viewed at https://wviechtb.github.io/mtafor/reference/influence.rma.uni.html. Table S2: Summary statistics from the meta‐regression used to assess the trend in the maximum rate of, and optimum temperature for photosynthesis of across latitude, but excluding Merimbula. Sensitivity diagnostics identified this site as exerting a strong influence on the model results. Removing this site did not change the statistical significance of any model parameters. Figure S3: Figures showing the trend in (a) the maximum [file ECE3-16-e73910-s001.pdf]

# 11 Supporting information

Table S1: Mean wet weight (± SE) of urchins used in metabolic experiments at each site.

| Species | Site | Wet weight (g ± SD) | Minimum wet weight (g) | Maximum wet weight (g) |
|---|---|---|---|---|
| *C. rodgersii* | Sawtell | 461.5 ± 53.3 | 389.6 | 561.9 |
| | Forster | 452.1 ± 184.0 | 302.6 | 843.7 |
| | Shellharbour | 366.3 ± 95.7 | 185.5 | 468.2 |
| | Merimbula | 381.8 ± 146.8 | 204.8 | 647.1 |
| | Mallacoota | 608.4 ± 130.6 | 402.2 | 827.2 |
| | Fortescue | 313.9 ± 97.3 | 147.7 | 485.4 |
| *H. erythrogramma* | Sawtell | 128.2 ± 27.2 | 91.7 | 172.6 |
| | Mallacoota | 269.7 ± 63.5 | 178.9 | 392.5 |
| | Forster | 197.8 ± 69.1 | 90.8 | 320.7 |
| | Shellharbour | 68.9 ± 12.1 | 57.7 | 94.0 |
| | Merimbula | 138.8 ± 28.9 | 99.3 | 196.3 |
| | Fortescue | 292.3 ± 46.0 | 224.0 | 341.2 |

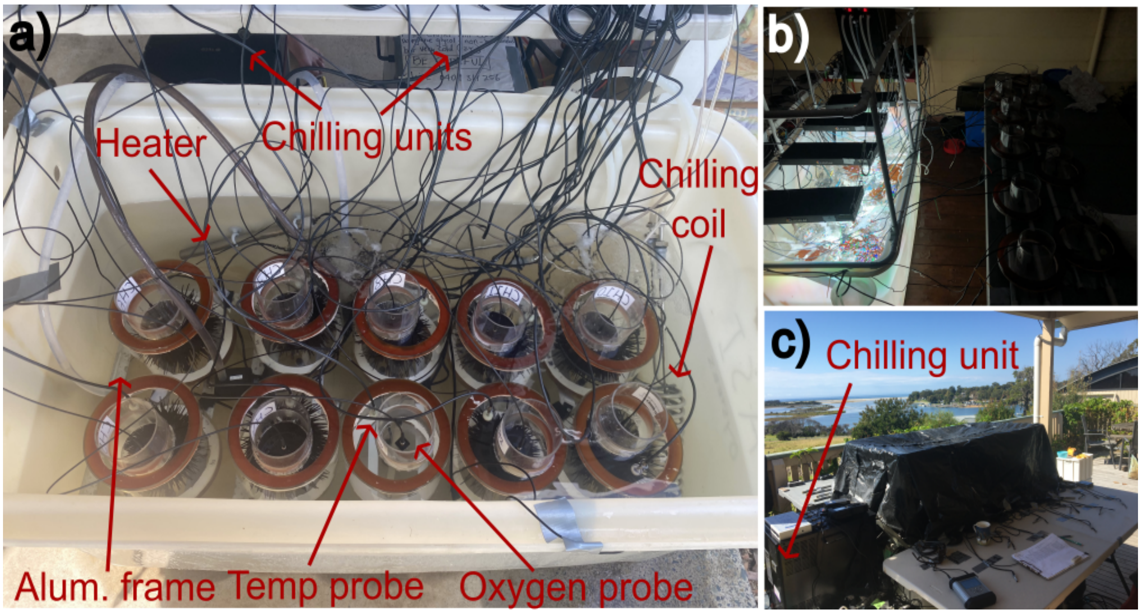


Figure S1: The custom-built experimental respiratory system. Shown are (a) ten 4.5L acrylic respiratory chambers, sitting on an aluminium frame housed within a 350L water bath. Each chamber has internal water circulation through pumps with inlet/outlet hose inserted through the base. Oxygen sensor spots are attached to the base of the lid, and oxygen probes aligned (via screw ports) directly above (within the chimney to avoid inundation). Temperature probes are inserted into each chamber through a port in the lid. One chamber is used as a control and allows measurement of background (microorganism) respiration. The water bath contains heating and chilling elements, and water pumps to ensure homogeneous temperature throughout. (b) To measure photosynthetic rates, a frame of plastic conduit was assembled and 4 LED lights were hung above the water bath. (c) The whole set-up was then covered in a black tarpaulin to to prevent additional daylight altering these standardised conditions.

Table S2: Treatment temperatures (°C) for each species at each site. Target temperatures were varied between locations and species in order to best capture the peak of the thermal performance curve.

| Site | *E. radiata* | *H. erythrogramma* | *C. rodgersii* |
|---|---|---|---|
| Sawtell | 6.5, 12.5 18.4, 22.5, 26.3, 30.2 | 6.4, 11.0, 15.7, 20.5, 25.1, 29.7, 35.0 | 7.5, 10.7, 15.8, 20.2, 25.2, 30.2, 35.0 |
| Forster | 5.6, 12.3, 18.3, 22.3, 26.2, 30.2 | 5.7, 12.5, 18.4, 22.2, 26.3, 30.1 | 5.2, 12.5, 18.5, 22.2, 26.1, 30.0, 33.7 |
| Shellharbour | 5.8, 12.3, 18.2, 21.3, 24.1, 27.3, 30.2 | 6.4, 12.5, 16.4, 21.4, 24.8, 27.0, 30.0 | 6.3, 12.2, 18.2, 22.0, 26.0, 30.0 |
| Merimbula | 5.9, 12.4, 18.3, 22.3, 24.2, 26.0, 28.0, 20.0 | 5.6, 12.4, 18.4, 21.4, 24.1, 26.9, 30.0 | 5.4, 12.3, 18.5, 21.1, 24.1, 27.0, 30.0 |
| Mallacoota | 5.2, 10.7, 17.2, 21.0, 23.1, 25.0, 28.1, 31.0 | 5.2, 12.2, 18.3, 21.1, 23.9, 26.9, 29.7 | 5.2, 12.3, 18.2, 21.1, 23.9, 26.8, 29.8 |
| Fortescue | 5.2, 9.2, 16.3, 20.4, 22.8, 24.8, 26.0, 29.9 | 5.2, 10.7, 17.4, 21.0, 23.9, 26.7, 29.5 | 5.1, 10.0, 17.1, 21.0, 24.0, 26.8, 29.8 |

Table S3: The dates on which the metabolic experiments were carried out for each species and location.

| Site | *E. radiata* | *C. rodgersii* | *H.erythrogramma* |
|---|---|---|---|
| Sawtell | 11/02/2023 | 08/02/2023 | 09/02/2023 |
| Forster | 18/02/2023 | 16/02/2023 | 19/02/2023 |
| Shellharbour | 24/02/2023 | 22/02/2023 | 23/02/2023 |
| Merimbula | 05/03/2023 | 02/03/2023 | 03/03/2023 |
| Mallacoota | 15/03/2023 | 12/03/2023 | 13/03/2023 |
| Fortescue | 24/03/2023 | 22/03/2023 | 21/03/2023 |

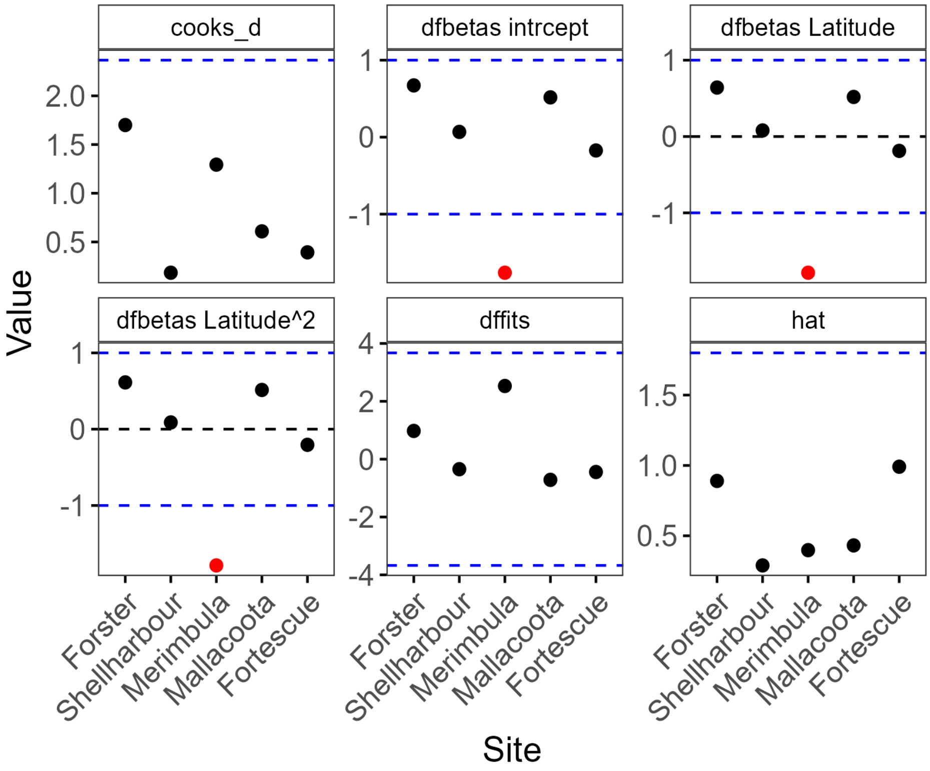


Figure S2: Leave-one-out model diagnostics for the maximum rate of photosynthesis of *E. radiata* across latitude. Plots show different computed influence metrics for each data point on the model fit. Thresholds specific to each influence metric are shown by blue dashed lines, and points outside these thresholds (i.e. those with high influence) are shown in red. An explanation of each of the influence metrics can be viewed at `https://wviechtb.github.io/metafor/reference/influence.rma.uni.html`

Table S4: Summary statistics from the meta-regression used to assess the trend in the maximum rate of, and optimum temperature for photosynthesis of *E. radiata* across latitude, but excluding Merimbula. Sensitivity diagnostics identified this site as exerting a strong influence on the model results. Removing this site did not change the statistical significance of any model parameters.

| Site excluded | Param | F-stat | DF | P-value | AIC |
|---|---|---|---|---|---|
| Merimbula | rmax | 72.723 | 2 | 0.000 | 1.705 |
| Merimbula | topt | 1.126 | 2 | 0.569 | 9.208 |

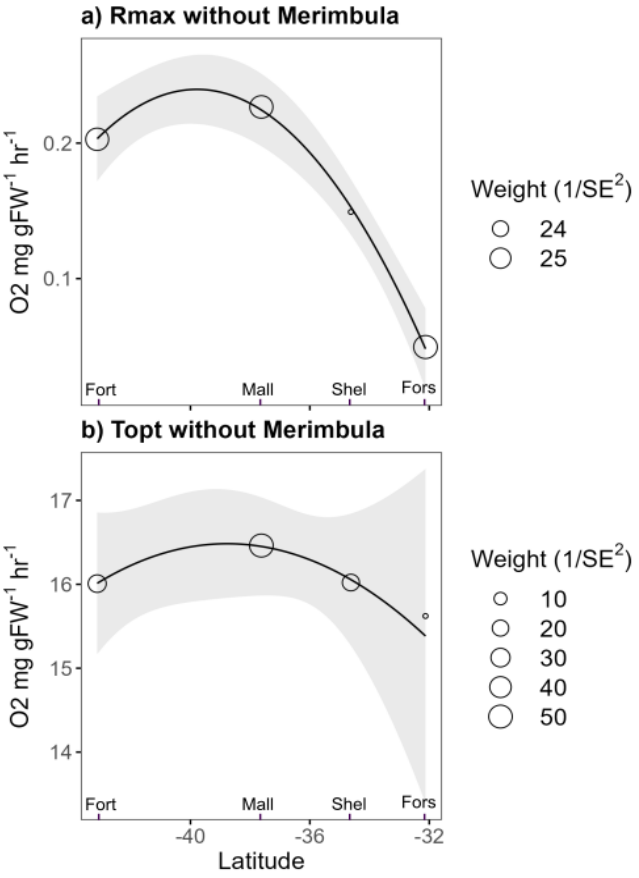


Figure S3: Figures showing the trend in a) the maximum rate of photosynthesis and b) the optimum temperature for photosynthesis of *E. radiata* across latitude, but excluding Merimbula from the model fitting. This site showed high influence on the model results. However, excluding Merimbula did not change the latitudinal pattern in $R_{max}$ or $T_{opt}$. Points in each panel represent the mean value of the given parameter with point size showing the inverse of the sampling variance. This indicates the weight given to each data point in the model, with larger sizes meaning the point was weighted more heavily (i.e. was more precise with lower standard error). In addition to excluding the parameter estimate from the highly influential site, the parameter values for the warmest site (Sawtell) were also not included in model fitting because there was no net gain in $O_2$ observed at this site.

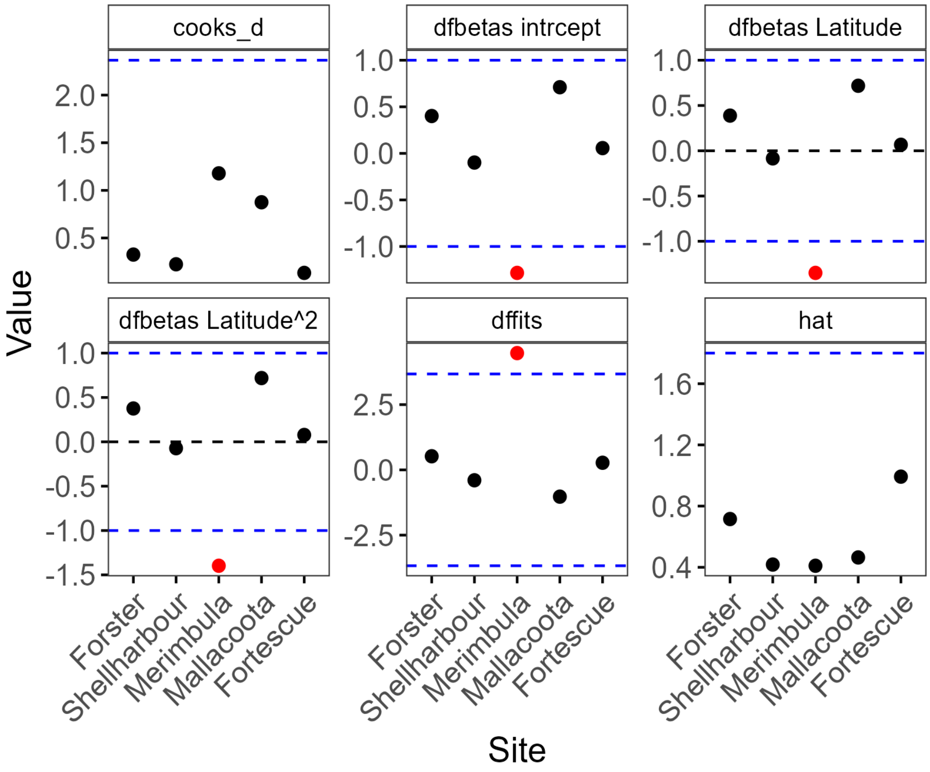


Figure S4: Leave-one-out model diagnostics for the optimum temperature for photosynthesis of *E. radiata* across latitude. Plots show different computed influence metrics for each data point on the model fit. Thresholds specific to each influence metric are shown by blue dashed lines, and points outside of these thresholds (i.e. those with high influence) are shown in red. An explanation of each of the influence metrics can be viewed at `https://wviechtb.github.io/metafor/reference/influence.rma.uni.html`

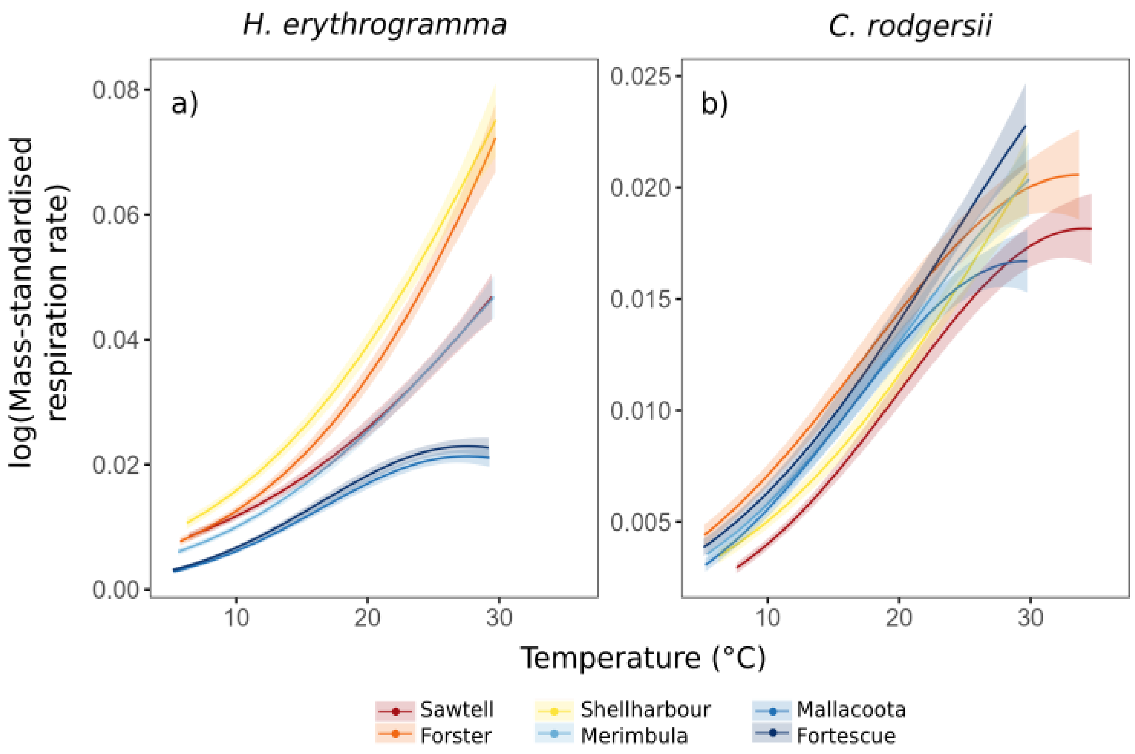


Figure S5: Mass-standardised respiration rates (mg $O_2$ $h^{-1}$) for (a) *H. erythrogramma* and (b) *C. rodgersii*, at each site across latitude.

Table S5: Results from the analysis of deviance for used to test the significance of the model fixed effects for mass-standardised respiration rates of *H. erythrogramma*.

| Fixed effect | Chisq | Df | Pr(>Chisq) |
|---|---|---|---|
| poly(temp, 2) | 7919.61 | 2 | 0.0000 |
| site | 176.91 | 5 | 0.0000 |
| poly(temp, 2):site | 141.69 | 10 | 0.0000 |

Table S6: A matrix showing the pairwise differences in estimated marginal means for mass-standardised respiration rates of *H. erythrogramma* at different sites across latitude. The matrix shows the estimated marginal means along the diagonal, the p-value in the upper triangle, and the differences between the estimated marginal means the lower triangle. Pairwise comparisons were carried out at the minimum (6.42 °C), median (17.7 °C) and maximum (29.2 °C) temperature of the shared temperature range that was tested across locations.

| Temp | Site | Sawtell | Forster | Shellharbour | Merimbula | Mallacoota | Fortescue |
|---|---|---|---|---|---|---|---|
| 6.42 | Sawtell | [-4.78] | 0.9994 | 0.2586 | 0.2426 | < 0.0001 | < 0.0001 |
| | Forster | 0.0372 | [-4.81] | 0.1230 | 0.4249 | < 0.0001 | < 0.0001 |
| | Shellharbour | -0.2407 | -0.2779 | [-4.53] | 0.0002 | < 0.0001 | < 0.0001 |
| | Merimbula | 0.2423 | 0.2051 | 0.4830 | [-5.02] | < 0.0001 | < 0.0001 |
| | Mallacoota | 0.8750 | 0.8378 | 1.1157 | 0.6327 | [-5.65] | 0.9997 |
| | Fortescue | 0.8436 | 0.8063 | 1.0843 | 0.6013 | -0.0314 | [-5.62] |
| 17.7 | Sawtell | [-3.82] | 0.2349 | 0.0015 | 0.9996 | 0.0010 | 0.0046 |
| | Forster | -0.2266 | [-3.59] | 0.5612 | 0.1152 | < 0.0001 | < 0.0001 |
| | Shellharbour | -0.3958 | -0.1692 | [-3.42] | 0.0004 | < 0.0001 | < 0.0001 |
| | Merimbula | 0.0318 | 0.2584 | 0.4276 | [-3.85] | 0.0030 | 0.0129 |
| | Mallacoota | 0.4068 | 0.6334 | 0.8026 | 0.3750 | [-4.23] | 0.9988 |
| | Fortescue | 0.3665 | 0.5931 | 0.7623 | 0.3347 | -0.0402 | [-4.19] |
| 29.2 | Sawtell | [-3.08] | 0.0042 | 0.0035 | 1.0000 | < 0.0001 | < 0.0001 |
| | Forster | -0.38857 | [-2.69] | 1.0000 | 0.0025 | < 0.0001 | < 0.0001 |
| | Shellharbour | -0.39617 | -0.00759 | [-2.68] | 0.0020 | < 0.0001 | < 0.0001 |
| | Merimbula | 0.00846 | 0.39703 | 0.40463 | [-3.09] | < 0.0001 | < 0.0001 |
| | Mallacoota | 0.79468 | 1.18326 | 1.19085 | 0.78622 | [-3.88] | 0.9939 |
| | Fortescue | 0.73587 | 1.12445 | 1.13204 | 0.72741 | -0.05881 | [-3.82] |

Table S7: Results from the analysis of deviance for used to test the significance of the model fixed effects for mass-standardised respiration rates of *C. rodgersii*.

| Fixed effect | Chisq | Df | Pr(>Chisq) |
|---|---|---|---|
| poly(temp, 2) | 4956.30 | 2 | 0.0000 |
| site | 18.45 | 5 | 0.0024 |
| poly(temp, 2):site | 68.89 | 10 | 0.0000 |

Table S8: A matrix showing the pairwise differences in estimated marginal means for mass-standardised respiration rates of *C. rodgersii* at different sites across latitude. The matrix shows the estimated marginal means along the diagonal, the p-value in the upper triangle, and the differences between the estimated marginal means the lower triangle. Pairwise comparisons were carried out at the minimum (7.62 °C), median (18.4 °C) and maximum (29.6 °C) temperature of the shared temperature range that was tested across locations.

| Temp | Site | Sawtell | Forster | Shellharbour | Merimbula | Mallacoota | Fortescue |
|---|---|---|---|---|---|---|---|
| 7.62 | Sawtell | [-5.86] | <.0001 | 0.0213 | 0.0005 | 0.0034 | <.0001 |
| | Forster | -0.6633 | [-5.19] | 0.0177 | 0.2029 | 0.0649 | 0.8966 |
| | Shellharbour | -0.3210 | 0.3423 | [-5.53] | 0.9207 | 0.9964 | 0.2198 |
| | Merimbula | -0.4209 | 0.2424 | -0.1000 | [-5.43] | 0.9960 | 0.8015 |
| | Mallacoota | -0.3707 | 0.2926 | -0.0498 | 0.0502 | [-5.48] | 0.4848 |
| | Fortescue | -0.5492 | 0.1141 | -0.2283 | -0.1283 | -0.1785 | [-5.31] |
| 18.4 | Sawtell | [-4.66] | 0.0277 | 0.9634 | 0.4475 | 0.3130 | 0.0483 |
| | Forster | -0.3081 | [-4.35] | 0.2134 | 0.7631 | 0.8684 | 0.9995 |
| | Shellharbour | -0.0784 | 0.2296 | [-4.58] | 0.9221 | 0.8329 | 0.3286 |
| | Merimbula | -0.1727 | 0.1354 | -0.0943 | [-4.49] | 0.9999 | 0.8973 |
| | Mallacoota | -0.1937 | 0.1143 | -0.1153 | -0.0210 | [-4.46] | 0.9599 |
| | Fortescue | -0.2744 | 0.0336 | -0.1960 | -0.1017 | -0.0807 | [-4.38] |
| 29.6 | Sawtell | [-4.06] | 0.8203 | 0.5953 | 0.8048 | 0.9972 | 0.0620 |
| | Forster | -0.1223 | [-3.94] | 0.9993 | 1.0000 | 0.5863 | 0.7007 |
| | Shellharbour | -0.1586 | -0.0363 | [-3.90] | 0.9994 | 0.3581 | 0.8828 |
| | Merimbula | -0.1230 | -0.0007 | 0.0356 | [-3.94] | 0.5637 | 0.6903 |
| | Mallacoota | 0.0460 | 0.1682 | 0.2046 | 0.1690 | [-4.11] | 0.0249 |
| | Fortescue | -0.2727 | -0.1505 | -0.1141 | -0.1497 | -0.3187 | [-3.79] |

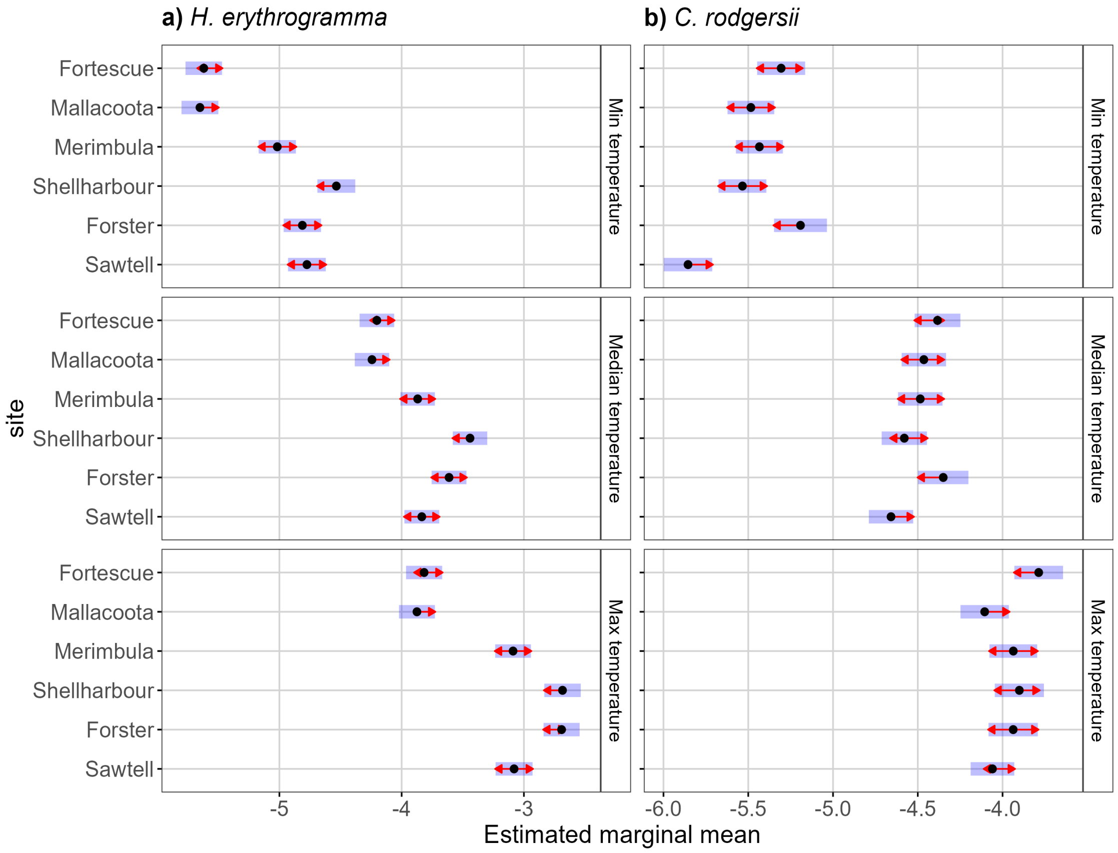


Figure S6: Pairwise differences in estimated marginal means for mass-standardised respiration rates of (a) *H. erythrogramma* and (b) *C. rodgersii* at different sites across latitude. Pairwise comparisons were carried out at the minimum, median and maximum temperature of the shared temperature range that was tested across locations. For *H. erythrogramma* this was 6.42 °C, 17.7 °C, 29.2 °C while for *C. rodgersii* these temperatures were 7.62 °C, 18.4 °C, 29.6 °C. Points show the estimated marginal mean, with 95% confidence intervals shown by the blue box. The red arrows show the pairwise comparison, with significant differences between groups represented by arrows that do not overlap.

Table S9: A matrix showing the pairwise differences in estimated marginal means for mass-independent respiration rates of *H. erythrogramma* at different sites across latitude. The matrix shows the estimated marginal means along the diagonal, the p-value in the upper triangle, and the differences between the estimated marginal means the lower triangle. Pairwise comparisons were carried out at the minimum (6.42 °C), median (17.7 °C) and maximum (29.2 °C) temperature of the shared temperature range that was tested across locations.

| Temp | Site | Sawtell | Forster | Shellharbour | Merimbula | Mallacoota | Fortescue |
|---|---|---|---|---|---|---|---|
| 6.42 | Sawtell | [−1.027] | 0.5702 | 0.8807 | 0.3819 | 0.0004 | 0.0110 |
| | Forster | −0.1751 | [−0.852] | 0.0662 | 0.0042 | <0.0001 | <0.0001 |
| | Shellharbour | 0.1186 | 0.2937 | [−1.146] | 0.9649 | 0.0251 | 0.2377 |
| | Merimbula | 0.2055 | 0.3806 | 0.0869 | [−1.233] | 0.1973 | 0.7314 |
| | Mallacoota | 0.4454 | 0.6205 | 0.3268 | 0.2399 | [−1.473] | 0.9418 |
| | Fortescue | 0.3504 | 0.5255 | 0.2318 | 0.1449 | −0.0950 | [−1.378] |
| 17.7 | Sawtell | [−0.0850] | 0.0001 | 0.9989 | 1.0000 | 0.9982 | 0.6125 |
| | Forster | −0.43676 | [0.3518] | 0.0006 | 0.0001 | 0.0007 | 0.0425 |
| | Shellharbour | −0.03757 | 0.39919 | [−0.0474] | 0.9994 | 1.0000 | 0.8368 |
| | Merimbula | −0.00486 | 0.43190 | 0.03271 | [−0.0801] | 0.9990 | 0.6388 |
| | Mallacoota | −0.04159 | 0.39517 | −0.00403 | −0.03673 | [−0.0434] | 0.8558 |
| | Fortescue | −0.15378 | 0.28298 | −0.11622 | −0.14892 | −0.11219 | [0.0688] |
| 29.2 | Sawtell | [0.668] | <0.0001 | 0.9994 | 0.9995 | 0.0058 | 0.2884 |
| | Forster | −0.59581 | [1.264] | <0.0001 | <0.0001 | <0.0001 | <0.0001 |
| | Shellharbour | −0.03545 | 0.56035 | [0.703] | 1.0000 | 0.0014 | 0.1358 |
| | Merimbula | −0.03363 | 0.56217 | 0.00182 | [0.701] | 0.0013 | 0.1344 |
| | Mallacoota | 0.36294 | 0.95874 | 0.39839 | 0.39657 | [0.305] | 0.7094 |
| | Fortescue | 0.21722 | 0.81303 | 0.25267 | 0.25086 | −0.14571 | [0.451] |

Table S10: A matrix showing the pairwise differences in estimated marginal means for mass-independent respiration rates of *C. rodgersii* at different sites across latitude. The matrix shows the estimated marginal means along the diagonal, the p-value in the upper triangle, and the differences between the estimated marginal means the lower triangle. Pairwise comparisons were carried out at the minimum (7.62 °C), median (18.4 °C) and maximum (29.6 °C) temperature of the shared temperature range that was tested across locations.

| Temp | Site | Sawtell | Forster | Shellharbour | Merimbula | Mallacoota | Fortescue |
|---|---|---|---|---|---|---|---|
| 7.62 | Sawtell | [−1.337] | <0.0001 | 0.0284 | 0.0003 | <0.0001 | <0.0001 |
| | Forster | −0.6922 | [−0.644] | 0.0002 | 0.0125 | 0.4706 | 0.0994 |
| | Shellharbour | −0.2794 | 0.4128 | [−1.057] | 0.8496 | 0.0642 | 0.4430 |
| | Merimbula | −0.3840 | 0.3082 | −0.1046 | [−0.953] | 0.5906 | 0.9826 |
| | Mallacoota | −0.5249 | 0.1673 | −0.2455 | −0.1409 | [−0.812] | 0.9500 |
| | Fortescue | −0.4460 | 0.2462 | −0.1666 | −0.0620 | 0.0789 | [−0.891] |
| 18.4 | Sawtell | [−0.11818] | 0.0027 | 1.0000 | 0.6999 | 0.0006 | 0.4972 |
| | Forster | −0.3178 | [0.19964] | 0.0069 | 0.1712 | 1.0000 | 0.3466 |
| | Shellharbour | −0.0164 | 0.3014 | [−0.10176] | 0.8290 | 0.0018 | 0.6484 |
| | Merimbula | −0.1158 | 0.2020 | −0.0994 | [−0.00239] | 0.0857 | 0.9994 |
| | Mallacoota | −0.3284 | −0.0106 | −0.3120 | −0.2126 | [0.21024] | 0.2149 |
| | Fortescue | −0.1437 | 0.1741 | −0.1273 | −0.0279 | 0.1847 | [0.02551] |
| 29.6 | Sawtell | [0.505] | 0.8084 | 0.9698 | 0.9964 | 0.9892 | 0.7394 |
| | Forster | −0.1044 | [0.609] | 0.9986 | 0.9814 | 0.9928 | 1.0000 |
| | Shellharbour | −0.0678 | 0.0365 | [0.572] | 0.9997 | 1.0000 | 0.9945 |
| | Merimbula | −0.0416 | 0.0628 | 0.0262 | [0.546] | 1.0000 | 0.9608 |
| | Mallacoota | −0.0531 | 0.0512 | 0.0147 | −0.0115 | [0.558] | 0.9813 |
| | Fortescue | −0.1180 | −0.0137 | −0.0502 | −0.0764 | −0.0649 | [0.623] |
